# Characterization of *Drosophila* GDNF Receptor-Like and Evidence for Its Evolutionarily Conserved Interaction with Neural Cell Adhesion Molecule (NCAM)/FasII

**DOI:** 10.1371/journal.pone.0051997

**Published:** 2012-12-20

**Authors:** Jukka Kallijärvi, Vassilis Stratoulias, Kristel Virtanen, Ville Hietakangas, Tapio I. Heino, Mart Saarma

**Affiliations:** 1 Institute of Biotechnology, Viikinkaari 9, Viikki Biocenter, University of Helsinki, Helsinki, Finland; 2 Department of Biosciences, Viikki Biocenter, University of Helsinki, Helsinki, Finland; Rutgers University, United States of America

## Abstract

**Background:**

Glial cell line-derived neurotrophic factor (GDNF) family ligands are secreted growth factors distantly related to the TGF-β superfamily. In mammals, they bind to the GDNF family receptor α (Gfrα) and signal through the Ret receptor tyrosine kinase. In order to gain insight into the evolution of the Ret-Gfr-Gdnf signaling system, we have cloned and characterized the first invertebrate *Gfr-like* cDNA (*DmGfrl*) from *Drosophila melanogaster* and generated a *DmGfrl* mutant allele.

**Results:**

We found that *DmGfrl* encodes a large GPI-anchored membrane protein with four GFR-like domains. In line with the fact that insects lack GDNF ligands, DmGfrl mediated neither *Drosophila* Ret phosphorylation nor mammalian RET phosphorylation. *In situ* hybridization analysis revealed that *DmGfrl* is expressed in the central and peripheral nervous systems throughout *Drosophila* development, but, surprisingly, *DmGfrl* and *DmRet* expression patterns were largely non-overlapping. We generated a *DmGfrl* null allele by genomic FLP deletion and found that both *DmGfrl* null females and males are viable but display fertility defects. The female fertility defect manifested as dorsal appendage malformation, small size and reduced viability of eggs laid by mutant females. In male flies *DmGfrl* interacted genetically with the *Drosophila* Ncam (neural cell adhesion molecule) homolog FasII to regulate fertility.

**Conclusion:**

Our results suggest that Ret and Gfrl did not function as an *in cis* receptor-coreceptor pair before the emergence of GDNF family ligands, and that the Ncam-Gfr interaction predated the *in cis* Ret-Gfr interaction in evolution. The fertility defects that we describe in *DmGfrl* null flies suggest that GDNF receptor-like has an evolutionarily ancient role in regulating male fertility and a previously unrecognized role in regulating oogenesis.

**Significance:**

These results shed light on the evolutionary aspects of the structure, expression and function of Ret-Gfrα and Ncam-Gfrα signaling complexes.

## Introduction

There is ample suggestive evidence that neurons in invertebrates require trophic support similarly to vertebrate neurons, although the identification of neurotrophic ligands in e.g. *Drosophila* has progressed only recently [1]. The first *Drosophila* homologs of vertebrate neurotrophin family proteins, *Drosophila* neurotrophin 1 (DNT1), DNT2 and Spätzle, were identified *in silico* several years ago [2] and recently characterized in detail and shown to possess neurotrophic activity *in vivo* [3]. Additionally, *DmManf*, the *Drosophila* homolog of the novel mammalian CDNF/MANF family of neurotrophic factors [4], is required for the development of the *Drosophila* embryonic nervous system [5].

Glial cell line-derived neurotrophic factor (GDNF) family ligands (GFLs) are secreted growth factors distantly related to the TGF-β superfamily [6], [7]. GFLs are crucial for the development and maintenance of distinct populations of central and peripheral neurons, as well as for the organogenesis of the kidney, and spermatogenesis. In mammals, four different GFL-coreceptor pairs exist. They all signal intracellularly through the RET receptor tyrosine kinase [6]. Neural cell adhesion molecule (NCAM) is an alternative signaling receptor for GDNF in mammals [8]. NCAM binds GFRα1 and GDNF and downregulates NCAM-mediated cell adhesion, which activates cytoplasmic protein tyrosine kinase signaling in the absence of RET. Through NCAM, GDNF stimulates Schwann cell migration and axonal growth in hippocampal and cortical neurons in mouse brain [8].

Mammalian GDNF family alpha receptors (GFRα) contain a conserved arrangement of extracellular cysteine-rich GFRα domains and a C-terminal GPI anchor [6]. Homologs of GFLs, RET and the four mammalian GFRα receptors exist in all vertebrates. RET homologs seem to be present in insects but not in echinoderms [9]. The *Drosophila melanogaster* RET homolog is expressed in many tissues analogous to the tissues where the gene is expressed in vertebrates, suggesting similar functions in development [10], [11]. GFR-like proteins have been identified *in silico* in sea urchin, insects and worms, including *D. melanogaster* and *C. elegans*. In *Drosophila*, two partial mRNA sequences encoding fragments of GFR-like proteins have been identified [9]. However, GDNF family ligand genes have not been found in invertebrates by *in silico* methods. To shed light on the evolutionary origin and function of invertebrate GFR-like proteins, we set out to characterize the *Drosophila melanogaster Gfr-like gene* (*DmGfrl*) gene and protein, to investigate its interaction with the mammalian GDNF receptors and to generate a *DmGfrl* null allele to investigate the *in vivo* functions of the receptor.

## Materials and Methods

### Fly Strains and Genetics

For most *in situ* hybridization experiments *w^1118^* flies were used. Embryos were staged according to Campos-Ortega and Hartenstein [12]. A *DmGfrl* gene trap line (FBti0126178) that harbors a PiggyBac insertion between exons 8 and 9 was obtained from Drosophila Genetic Resource Center. The genomic deficiency lines *Df(3R)Exel6185* (FBab0038240; referred to from this point on as *Df1*) and *Df(3R)BSC518* (FBab0045364, referred to from this point on as *Df2*) were obtained from Bloomington Drosophila Stock Center. For the generation of transgenic fly lines, *V5-DmGfrlA* and *DmRet-3xFLAG-6xHis* cDNAs was subcloned into pUAST. Transgenic lines were generated at Genetic Services, Inc. (Cambridge, MA, USA) or at Fly Facility, Inc. (Clermont-Ferrand Cedex, France). Transgene insertion chromosomes were mapped and balanced stocks generated. The *tyrosine hydroxylase* (*TH*)-GAL4 driver line was originally generated in the laboratory of Serge Birman [13]. The *tubulin*, *daughterless*, *elav*, *nubbin*, *GMR* and *eyeless* driver lines were from Bloomington Drosophila Stock Center. The dMP2-Gal4 line was a gift from Dr. Irene Miguel-Aliaga (Cambridge, UK). The *FasII^e76^* line was a gift from Dr. Mathew Freeman (Cambridge, UK).

To generate a *DmGfrl* null allele, FLP deletion between two PBac insertions was performed essentially as described [14]. Briefly, fly lines carrying the insertions *PBac{WH}f03437* and *PBac{WH}mun[f00705]* (Exelixis collection, Harvard) were both crossed with a line carrying hs-FLP transgene on 1st chromosome and 3^rd^ chromosome balancers. The resulting progeny were crossed to get the PBac elements *in trans* on 3^rd^ chromosome. Larvae from this cross were heat shocked in a water bath at 37°C for 1 hour on four consecutive days. Virgin females were collected from the heat-shocked progeny and crossed with *Tm3/Tm6B* balancer males. From the resulting progeny individual males were collected and crossed again with *Tm3/Tm6B* balancer females. After larvae were visible in the vials the males were removed and genotyped by PCR. The genotyping primers were.

WH5′plus TCCAAGCGGCGACTGAGATG,

WH3′plus CCTCGATATACAGACCGATAAAAC,

WH3′GP-fw GCCGTTGTTATTGCGACTTT and

WH5′GP-rw CGAAATGCGGACTTCAATTT.

Individuals harboring both PBac elements on the same chromosome were interpreted as having recombined successfully. Balanced stocks were established from PCR positive lines.

### RACE, RT-PCR and Construction of Expression Plasmids

Primers for Rapid Amplification of cDNA Ends (RACE) analysis were designed on the basis of a previously annotated *DmGfrl* cDNA fragment (Genbank accession no. NM_001014642). RACE analysis was performed using the Generacer kit (Invitrogen) and total RNA isolated from stage 1–17 *Drosophila* embryos. The transcript structures were verified by sequencing RT-PCR fragments spanning the entire predicted coding regions of the cDNAs. For expression studies, *DmRet* coding region was amplified by PCR from embryo cDNA using Phusion polymerase (Finnzymes) and subcloned into the pMT-A vector (Invitrogen) in frame with C-terminal V5 and hexahistidine tags. Plasmids encoding C-terminally 3xFLAG-tagged DmRet and N-terminally V5-tagged DmGfrl and were constructed by means of inverse PCR mutagenesis. Expression plasmids in which the nucleotides coding for the predicted native signal sequence of DmGfrl were either deleted or replaced by *Drosophila* luminal binding protein (BiP) signal sequence, and constructs in which the putative C-terminal GPI anchoring sequence (28 C-terminal amino acids) was deleted, were constructed by means of PCR. For expression in mammalian cells *DmGfrlA* cDNA was subcloned into pcDNA6/V5-His (Invitrogen) with IgGκ signal sequence.

### Northern Blotting

Total RNA was extracted from *w^1118^* embryos, larvae, pupae and adult flies either by the TRIZOL reagent (Gibco BRL, Life Technologies). Poly-A-mRNA was enriched using the NucleoTrap mRNA kit (Macherey-Nagel) or oligo-dT cellulose (Calbiochem). Digoxigenin-labelled RNA probes were synthesized by *in vitro* transcription according to a standard protocol. The *DmGfrl* coding region probes used in both Northern blotting and *in situ* hybridization correspond to nucleotides (the numbering is relative to the ATG codon of transcript A) 147–1092 (in exons 5–12) and 1147–1841 (in exons 11–17). Moreover, 5′UTR probes specific for transcripts A and Ab (exons 4–5), and for transcripts B and Bb (exons 1–3) were used. The template for the preparation of the probe for the ribosomal *protein 49* (*rp49*) was amplified by PCR from adult fly cDNA with primers incorporating a T7 primer sequence to the 3′ terminus of the PCR fragment. A *DmRet* probe corresponding to the full-length coding sequence (3708 nt) was used in Northern blotting and a 567-nt probe corresponding to the 3′ end of the coding region in the *in situ* hybridizations. For Northern blot analysis, 1.5 µg of poly(A) RNA per lane was separated on 1% or 0.8% agarose-2% formaldehyde-MOPS gel. Blots were hybridized with digoxigenin-labelled RNA probes according to the manufacturer’s protocol (Roche). Label was detected using alkaline phosphatase-coupled digoxigenin antibody (Roche) and the chemiluminescent CSPD substrate (Roche).

### In situ Hybridization and Immunostaining

RNA *in situ* hybridization to embryos was performed either according to a standard protocol [15] or using a modified protocol without proteinase K treatment [16]. Hybridization was performed at 60°C for 16–18 hours. Third instar larval brains and adult fly brains were dissected and immediately fixed in 4% PFA in for 15 minutes and 30 minutes, respectively. *In situ* hybridization to the larval and adult brains was performed essentially as described elsewhere [17]. Images of adult brains were collected using a Nikon eclipse 90i microscope with a Nikon DS-5Mc camera.

For immunofluorescence staining, S2 cells transcfected with various *DmGfrl* and *DmRet* expression constructs were allowed to attach to concanavalin A (Sigma)-coated glass coverslips for 1 hour. The cells were then fixed with 3.7% formaldehyde in PBS for 20 minutes. The cells were either permeabilized with 0.5% NP-40 in PBS or left unpermeabilized, and incubated with a V5 tag antibody (Invitrogen), followed by a Cy2-conjugated secondary antibody (Jackson ImmunoResearch Laboratories). Imaging was performed with an Olympus AX70 epifluorescence microscope. The following antibodies were obtained from Developmental Studies Hybridoma Bank at the University of Iowa and used for embryo immunostainings: anti-Repo (mAb 8D12), anti-FasII (mAb 1D4), anti-prospero (MR1A), anti-cut (2B10), anti-Futsch (22C10). Additionally, a rabbit anti-β-galactosidase antibody (Cappel) was used. Whole mount embryos were examined under an Olympus AX70 microscope and images were taken by Olympus DP70 camera. Immunoperoxidase staining of embryos was performed using Vectastain ABC reagents (Vector Laboratories Inc.) according to standard methods.

### Generation of Anti-DmRet and Anti-DmGfrl Antibodies

GST fusion proteins comprising amino acids 1046–1235 of DmRet and amino acids 649–958 of DmGfrlA were produced in *E. coli*, solubilized with 0.7% N-lauroylsarcosine and purified with glutathione sepharose beads (GE Healthcare). Immunization of rabbits with the soluble GST-antigen fusion proteins were carried out according to standard methods by Inbiolabs Inc., Tallinn, Estonia. Specificity of the final antisera in immunoblotting and immunoprecipitation were determined against epitope-tagged proteins expressed in S2 cells.

### Cell Culture and Biochemical Assays

Schneider 2 (S2) cells were cultured in M3-BPYE medium (Shields and Sang M3, 0.5 g/l KHCO_3_, 1.0 g/l yeast extract, 2.5 g/l bactopeptone and 10% fetal bovine serum, pH 6.6) at +25°C. Transfections were performed using Fugene HD reagent (Roche). Expression from the metallothionein promoter of pMT was induced with to 600 µM CuSO_4_. Three days post-transfection, the cells were washed once with PBS and lysed in membrane lysis buffer (TBS, 1% Triton X-100, 20 mM NaF, 1 mM EDTA, pH 7.5) containing proteinase inhibitor cocktail (Roche). Insoluble material was sedimented by centrifugation and the soluble fraction was analyzed by SDS-PAGE and immunoblotting. Stably transfected S2 cell lines inducibly expressing DmRet-3xFLAG or DmRet-3xFLAG and V5-DmGfrlA were generated by cotransfecting S2 cells with the appropriate expression constructs and pCoHygro. Stable pools were selected with 300 µg/ml and maintained in 100 µg/ml hygromycin. For coimmunoprecipitation experiments S2 cells were transfected in 6-well plates and expression was induced for 2 to 3 days. The cells were collected and lysed in Co-IP buffer (TBS, 1% Triton X-100, 20 mM NaF, 1 mM dithiotreitol, 0.9 mM CaCl_2_, 0.5 mM MgCl_2_). Immunoprecipitation was performed with 0.5 µg of affinity-purified DmRet or DmGfrl antibodies or tag antibodies.

For detection of endogenous DmGfrl, 25 adult flies per genotype were homogenized in 50 mM octyl glucoside in TBS containing proteinase inhibitors, calcium and magnesium. Insoluble material was sedimented and the resulting supernatants were precleared with plain sepharose beads. To the precleared lysates a mixture of peanut agglutinin- and jacalin-conjugated agarose beads (20 µl) was added and the mixtures were rotated for 1 h in cold. The beads were washed with the lysis buffer, followed by elution with Laemmli buffer, 8% SDS-PAGE and immunoblot analysis with the DmGfrl antiserum (1∶5000).

For the digestion of cell surface GPI-anchors, ∼1 million S2 cells transfected with a DmGfrlA expression construct were washed twice in PBS and then digested in 50 µl of PBS with 0.4 U of phosphatidylinositol-specific phospholipase C (PI-PLC, Sigma) at +4°C for 20 minutes. The cells were then sedimented at 700 g for 1 minute and the supernatant recovered. The cellular and soluble fractions were analyzed by immunoblotting.

For DmRet phosphorylation assays, cells stably expressing DmRet-3xFLAG or DmRet-3xFLAG and V5-DmGfrlA were plated in 6-well plates at ∼12 M cells/well, in serum-free M3-BPYE medium. Expression was induced with 600 µM CuSO_4_ for 2 to 4 hours. Recombinant human GDNF (PeproTech Ltd.) was added at 50 ng/ml for 1 hour after which the cells were triturated, washed in PBS, and lysed in membrane lysis buffer supplemented with 1 mM Na_2_VO_4_. Insoluble material was sedimented by centrifugation and to the supernatants 0.5 µg of affinity-purified DmRet antibody was added. After 15-minute incubation on ice, protein A-sepharose (GE Healthcare) was added and the samples were rotated in cold for 1–2 hours. The precipitates were washed with the lysis buffer three times, eluted with Laemmli sample buffer, and run on 8% SDS-PAGE. The immunoblots were probed with anti-phosphotyrosine (4G10, Millipore) and anti-FLAG (M2, Sigma) antibodies. For mammalian RET phosphorylation assay MG87RET cells that stably express human RET51 were transfected with plasmids encoding human GFRα1 or DmGfrlA. RET phosphorylation assay was performed essentially as described previously [18].

For heparin binding assay, GPI-deleted DmGfrlA was expressed in S2 cells and 10 ml cell culture medium collected. The medium was rotated with 500 µl of heparin-sepharose beads (GE Healthcare) for 1 hour. The beads were packed in a column and washed with 10 mM HEPES pH 7.2, 0.1 M NaCl. Fractions of 250 µl were eluted with 0.2 to 1.2 M NaCl in HEPES pH 7.2. The fractions were analyzed by immunoblotting using a V5 tag antibody. To assess the heparin binding of DmGfrl *in vivo*, adult transgenic flies expressing V5-DmGfrl in the nervous system (*elav-GAL4/Sm;V5-DmGfrl/Tm*) were collected (50 adults). The flies were homogenized in 50 mM octyl glucoside in TBS and insoluble material was sedimented. The lysates were precleared with plain sepharose beads and then rotated with 100 µl of heparin-sepharose beads in the cold. The beads were washed with the lysis buffer and bound proteins were eluted with a NaCl gradient in lysis buffer. The eluates were analyzed by immunoblotting using V5 tag antibody.

### Fertility and Egg-laying Assays

To image and quantify the morphology of eggs laid by *DmGfrl* mutant females, five females of the appropriate genotypes were placed in small vials with five *w^−^* males and some yeast paste and allowed to mate for 24 hours. After this, the flies were placed in small chambers on apple juice plates with some yeast paste and allowed to lay egg for ∼20 hours. The number of normal and malformed (small size and/or abnormal dorsal appendages) eggs per plate were counted under a stereomicroscope blindly with respect to the genotype. The eggs were imaged with ProgRes SpeedXT camera (Jenoptik) and egg lengths measured with ProgRes Capture Pro software (Jenoptik). The images were processed with Adobe Photoshop CS5 software. Paint and erase tools were used to remove and correct uneven background resulting from rotation and moving of the eggs for better presentation. For egg length quantification, ∼30 eggs per genotype were placed horizontally on apple juice plates under a stereomicroscope and imaged as above. One-way ANOVA with Tukey’s post hoc test was used in statistical analysis of the egg length data and non-parametric Kruskal-Wallis ANOVA with Dunn’s post hoc test in that of the rescue data.

For male fertility and fecundity quantification, individual males were placed in small vials with three wild-type (*w*) virgin females (aged for at least two days) and some dry yeast. Visible larvae were counted after 72 hours at 25°C. For female fertility and fecundity quantification, individual females (aged for at least two days) were placed in small vials with three wild-type (*w*) males and some dry yeast. Visible larvae were counted after 72 hours. Fertility was defined as the percentage of vials with viable larvae. Fecundity percentage was calculated as the mean number of larvae per vial per genotype divided by the mean number of larvae per vial for the control genotype (*del/+*). Mutant males and females less than one week old were mainly used in the assays and they were mated with *w^−^* males or virgin females less than 2 weeks old. Only those vials that had visible eggs and at least 2 of the 3 wild-type males/females alive at 72 h were scored. Altogether 10–20 individuals per genotype were scored. One-way ANOVA with Bonferroni’s post hoc test was used for statistical analysis of the male fertility data.

## Results

### The *Drosophila GDNF Receptor-like* (*DmGfrl*) cDNA Encodes a Large Protein with Four Cysteine-rich GFRα-like Domains

**Figure 1:**
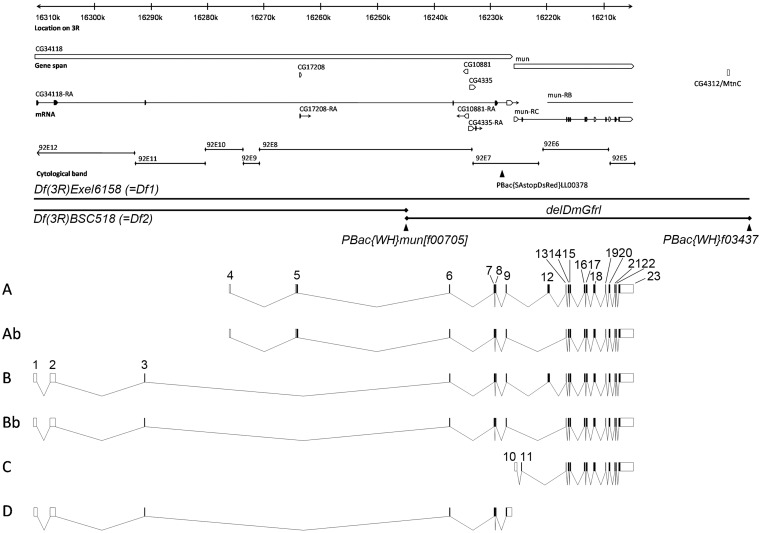
Genomic and transcript structure of the *DmGfrl* locus. A 105-kb region of chromosome 3R is shown. The annotation is according to Flybase version FB2009_07, released August 10, 2009. On the basis of our cDNA cloning and sequencing results, the currently annotated *mun*/*DmGfrl* locus is extended into the 5′ direction by approximately 85 kb, and the genes previously annotated as CG34118 and CG17208 are fused with *DmGfrl*. CG4335 and CG10881, predicted intronless genes encoding a trimethyllysine dioxygenase and a translation initiation factor, respectively, are embedded on opposite strands in the 7.5-kb intron between exons 6 and 7. The Flybase entries previously annotated as CG17208 and CG34118 were identified as being the 5′ termini of the *DmGfrl* transcripts A and B, respectively. *PBac{SAstopDsRed}LL00378* is a hypomorphic insertion utilized in the biochemical detection of endogenous DmGfrl protein (see Fig. 4). *Df(3R)Exel6185* (*Df1*) and *Df(3R)BSC519* (*Df2*) are the genomic deficiencies used in the genetic experiments. *delDmGfrl* denotes the deletion allele generated by means excision between the FRT-carrying PBac elements *PBac{WH}mun[f00705]* and *PBac{WH}f03437*. Exon-intron structures of the six *DmGfrl* transcripts (A, Ab, B, Bb, C, D) assembled from experimental and *in silico* data are presented below the genomic locus drawing. Transcripts C and D were not detected on Northern blots, which suggest that they are present at very low levels compared to the other transcripts, and thus, their physiological significance remains to be investigated.

**Figure 2:**
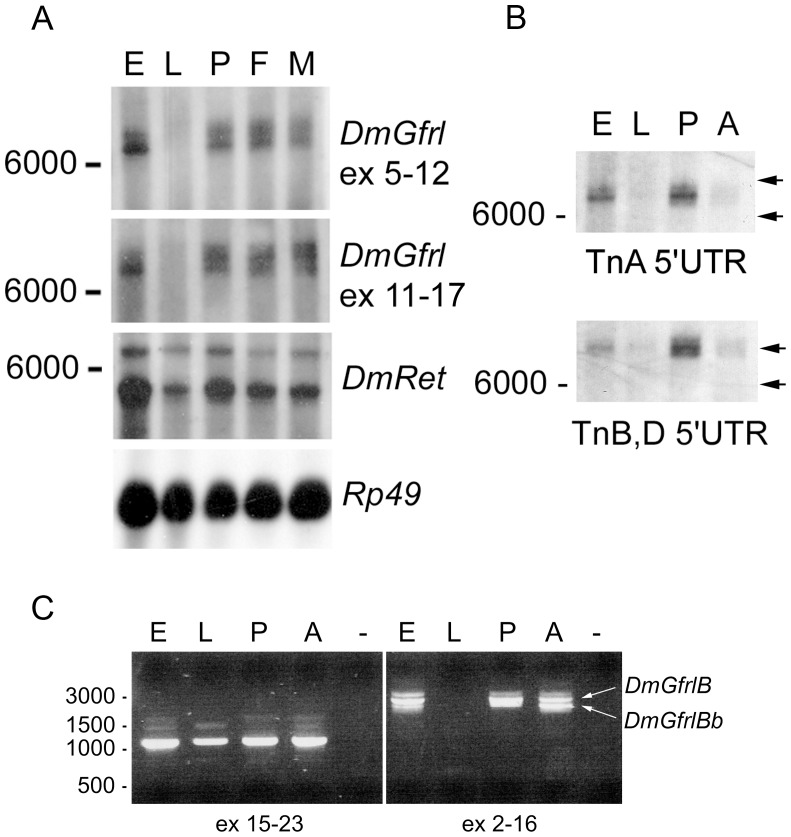
Northern blot and RT-PCR analysis of *DmGfrl* and *DmRet* mRNA expression. (A) Two transcripts of approximately 7000 and 7500 nucleotides were detected in embryonic (E), pupal (P), adult female (F) and adult male (M) stages, but not in 3rd instar larvae (L). An essentially identical transcript pattern was detected with a probe corresponding to a region coding for the first two GFRα-like domains (exons 5–12) and with a probe corresponding to a region coding for the latter two GFRα-like domains (exons 11–17). With a *DmRet* probe, a major band of ∼5000 nt and a weaker band of ∼6500 nt were detected at all developmental stages. Hybridization for the rp49 gene showed roughly equal poly-A RNA loading. (B) Hybridization with 5′ untranslated region (UTR) probes revealed that the two major *DmGfrl* mRNA bands (∼7000-nt and ∼7500-nt) arise from differential transcription start site usage, with the ∼7000-nt band corresponding to transcript A and the ∼7500-nt band correcponding to transcripts B. The calculated size difference between the two 5′UTRs is ∼590 nt. Note that the 5′UTR probes give a markedly weaker signal in adults (A) than in pupae (P), which may be due to alternative splicing of the 5′ non-coding exons in adult tissues. Equally spaced arrows on the right illustrate the size difference of the two transcripts. (C) In RT-PCR with primers designed to amplify a 1085-bp fragment from exons 15–23 of *DmGfrl* one major major DNA band was detected in embryonic (E), larval (L), pupal (P) and adult (A) tissues (left image). With primers designed to amplify the entire coding region of *DmGfrl* transcript B, two bands of approximately 2.3 kb and 2.6 kb were detected in adult tissues (right image). The 2.3-kb band was sequenced and found to correspond to an alternative transcript (named Bb) that lacks exon 12. The lower band was weaker in embryonic tissues, suggesting developmentally regulated splicing. In pupal tissues a lower band barely separated from the 2.6-kb band is seen (right panel). This band may represent additional alternative splicing in this tissue. The faint bands above the major *DmGfrl* band in the left panel likely represent partially spliced mRNAs.

**Figure 3:**
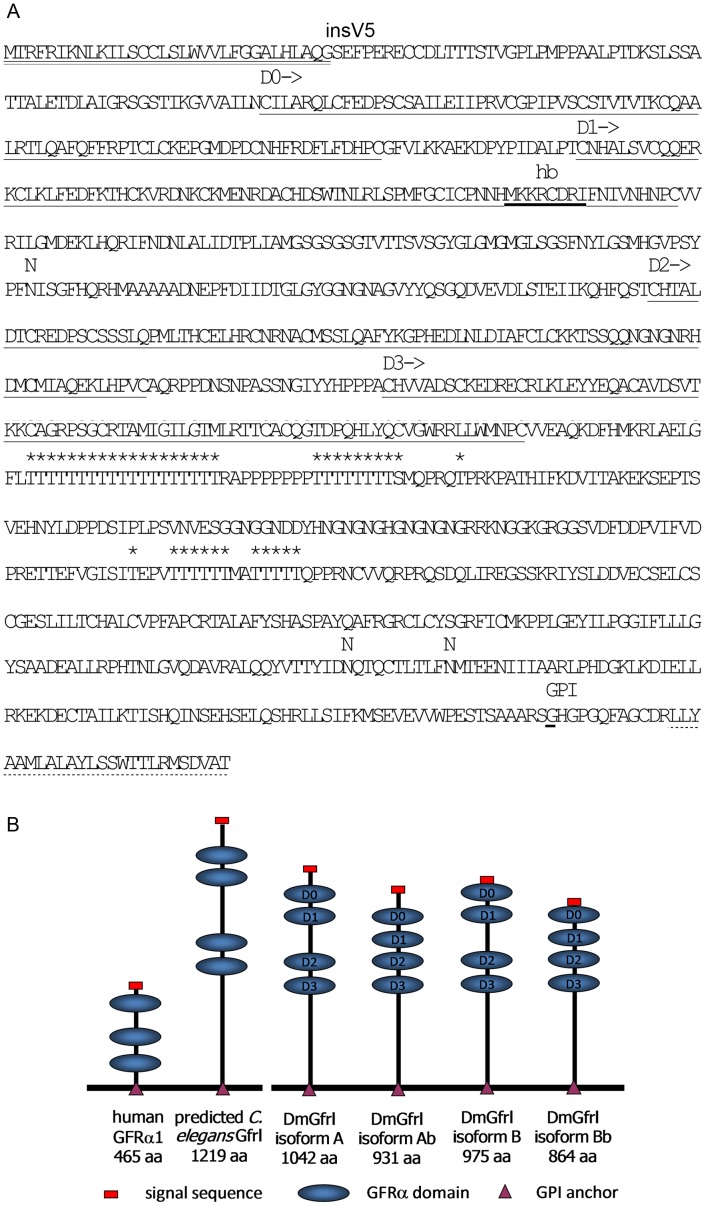
Amino acid sequence of DmGfrlA and schematic structures of the predicted DmGfrl protein isoforms identified in this study. (A) In the amino acid sequence of DmGfrl protein isoform A the signal sequence is marked with solid double underlining. The amino acid sequence of isoform B is identical to that of isoform A except for 26 N-terminal amino acids preceding the D0 domain, which reads MLKPFAVIIGIFYLGSTIKGVVAILN in DmGfrlB. V5 after the signal sequence denotes the site (between Q31 and G32) where a sequence encoding the V5 tag (GKPIPNPLLGLDST) was inserted in some of the expression constructs. The GFRα-like domains 0 to 3 (D0 to D3) are marked with solid underlining and the sequence (MKKCDRI) similar to mammalian heparin binding sites with bold underlining. The three predicted N-glycosylation sites are marked with N above the amino acid sequence and the mucin-type glycosylation sites with asterisks (*). A predicted low-score (big-PI Predictor) GPI anchoring site (G1008, underlined) is marked with ‘GPI’. It precedes a C-terminal hydrophobic region typical of GPI anchored proteins (dash underlining). (B) Schematic structures of four predicted DmGfrl isoforms and comparison to human GFRα1 and predicted *C. elegans* Gfr-like protein. Several insect genomes encode Gfr-like proteins that are approximately double the size of vertebrate Gfrα proteins [30]. Most of this size difference is due to the long sequence between the fourth GFRα-like domain and the plasma membrane anchor in the insect proteins.

Because full-length *DmGfrl* cDNA had not been characterized we set out to search experimentally for 5′ exons containing a consensus translational initiation site and encoding a putative signal sequence, as well as to uncover the transcript structure of the whole locus. The genomic structure of *DmGfrl* (Fig. 1) was assembled by BLASTing the experimentally resolved cDNA sequences and cDNA sequences derived from the Genbank against the *Drosophila* genome sequence. In the RACE analysis, starting from the existing EST fragments, a putative 5′ cDNA terminus with a consensus Kozak sequence, an ATG codon and multiple upstream stop codons were identified (data not shown). Another putative 5′terminus was identified in BLAST analysis of Genbank EST sequences and corresponded to the cDNA clone GH26447. A single putative 3′ cDNA terminus corresponding to an EST clone (now removed as a result of standard genome annotation processing, but identical with part of the clone MIP08659) was identified by BLAST analysis. With PCR primers designed for the two putative 5′ termini and the single putative 3′ terminus, RT-PCR amplified ∼3.1-kb (Fig. 2C) and ∼2.9-kb (data not shown) fragments from embryonic, larval and adult fly mRNA (data not shown). Upon sequencing, the PCR products were found to contain open reading frames of 3129 and 2928 base pairs, respectively. We named these transcripts A and B, respectively. In adult flies, RT-PCR revealed two additional transcript variants that were, upon sequencing, found to lack a single exon (exon 12) but were otherwise identical to transcripts A and B (Fig. 2C). We named these transcripts Ab and Bb, respectively. A total of 23 exons that were spread over 105 kb of genomic DNA were identified. Exon 1 starts at approximately 16.310 Mb and exon 23 ends at approximately 16.205 Mb on chromosome 3R, which corresponds to the cytological position 92E12-92E5 (Fig. 1). We identified two additional transcript variants (C and D, see Fig. 1) in EST sequences in Genbank and confirmed their presence in *Drosophila* tissues by RT-PCR (data not shown). Schematic structures of the predicted DmGfrl isoforms A, Ab, B and Bb are presented in Fig. 3B, and a summary of the *DmGfrl* exons identified or verified in this study in Table S1. Alignment of the GFRα-like domains of DmGfrl with each other and of DmGfrl domain 2 with GFRα-like domains from various invertebrates and vertebrates are shown in Fig. S1.

Northern blot and RT-PCR analyses revealed that *DmGfrl* mRNA is present at all developmental stages, although at very low levels in larvae (Fig. 2). In Northern blots, two transcripts of ∼7000 and ∼7500 nucleotides were detected in embryos, pupae and adult flies. The mRNAs were barely detectable in the 3rd instar larvae (Fig. 2A). Bands corresponding to the predicted sizes of the *DmGfrl* transcripts C and D were not detected on the blot, which suggests that these transcripts are present at very low levels. Hybridization with a *DmRet* probe showed a major transcript of ∼5000 nt in all developmental stages (Fig. 2A), which is in line with the report showing a 4.8-kb *DmRet* mRNA species in stage 1–17 embryos [10]. Hybridization with probes corresponding to the two *DmGfrl* 5′ termini identified by RACE and BLAST analysis revealed that the two main bands correspond to transcripts A and B, with a size difference of ∼600 nt arising from different 5′ UTRs (Fig. 2B).

According to *in silico* protein prediction and primary structure analysis the major DmGfrl isoforms A and B have a predicted N-terminal signal sequences and contain four cysteine-rich domains (D0 to D3) homologous to the mammalian GFRα domains. The theoretical molecular weight of DmGfrlA with the predicted signal sequence is 114.2 kDa and pI 6.81. The protein has three predicted N-glycosylation sites, at asparagines 343, 918 and 928 (NetNGlyc 1.0). Digestion of DmGfrl immunoprecipitates with PGNaseF modestly lowered the apparent molecular weight of both protein species, suggesting that DmGfrl is indeed N-glycosylated (our unpublished data). Moreover, 38 threonine residues in the threonine stretches at amino acids 615–777 are predicted as high-score mucin-type O-glycosylation sites (NetOGlyc 3.1, [19]). All isoforms share the C-terminus, which has a short hydrophobic stretch similar to GPI anchoring sequence [20]. Interestingly, a sequence similar to mammalian heparin binding sites [21] is present in domain 1 (Fig. 3A, marked as ‘hb’). The amino acid sequence of DmGfrlA, the locations of the GFRα-like domains and the predicted post-translational modification sites are depicted in Fig. 3A.

### The DmGfrl Protein is Secreted, Glycosylated and GPI Anchored on the Plasma Membrane

**Figure 4:**
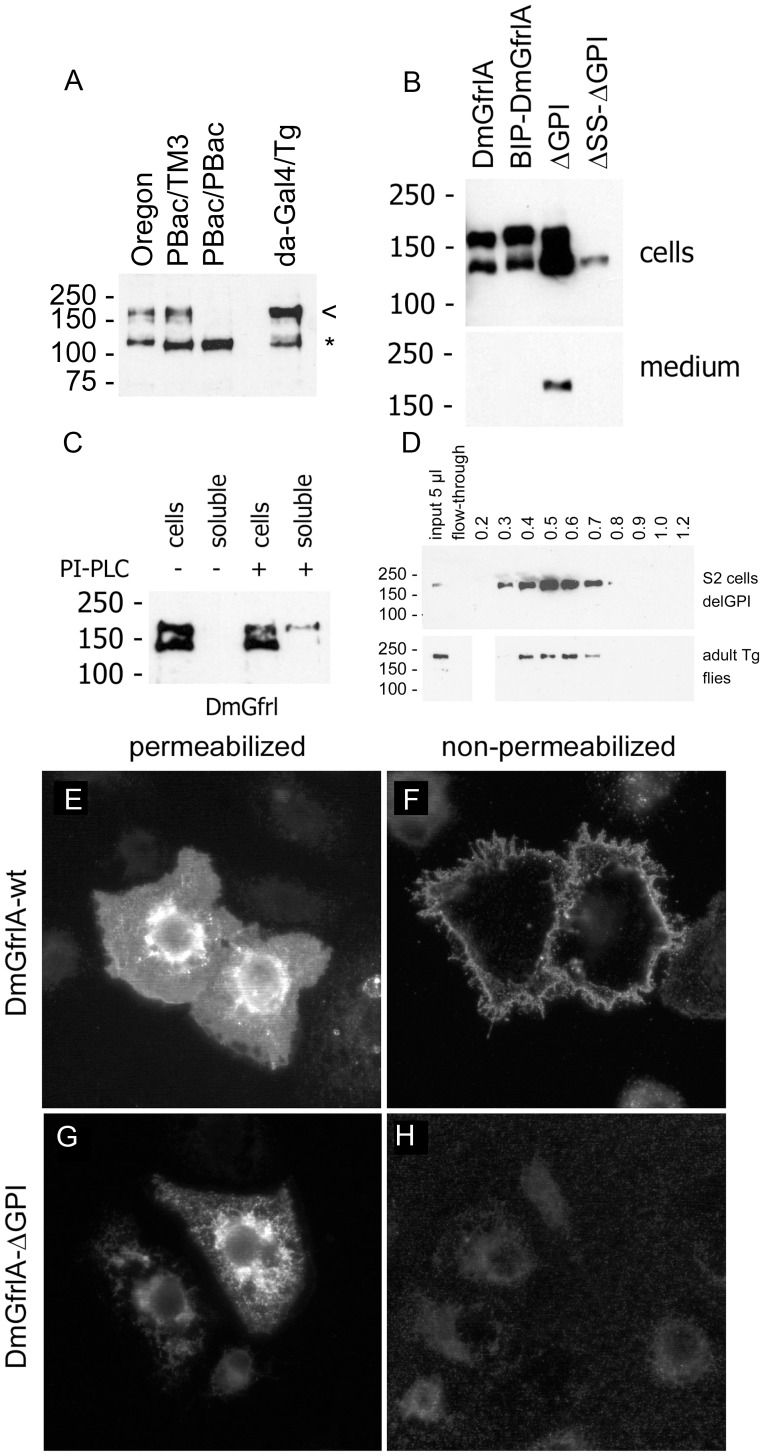
Detection and biochemical characterization of the DmGfrl protein. (A) Detection of endogenous DmGfrl by immunoblotting in lectin-enriched fractions from whole adult flies. Transgenic flies expressing DmGfrlA (*da-GAL4xTg*) show a band of ∼170 kDa (a, arrowhead), compatible with the predicted molecular weight of the ectopic protein. In wild-type (Oregon) flies a protein species of similar molecular weight was detected. The ∼170-kDa band was also present in heterozygous (A, *PBac/TM3*) *DmGfrl* genetrap flies, but absent in homozygous (A, *PBac/PBac*) genetrap flies that are *DmGfrl* hypomorphs (our unpublished data). Asterisk denotes a non-specific band. (B) Secretion of DmGfrlA. In S2 cells transfected with N-terminally V5-tagged DmGfrlA expression plasmids bands of ∼140 and ∼170 kDa were detected (B, 1st lane). The upper and lower bands likely represent fully glycosylated cell-surface-exposed protein and partly (core N-) glycosylated immature protein being synthesized and modified in the endoplasmic reticulum/Golgi, respectively. The wild-type protein as well as a mutant in which the putative N-terminal signal sequence was replaced by a known signal sequence (BIP, B, 2nd lane) were detected exclusively in the cellular fraction, whereas a mutant protein lacking the predicted C-terminal GPI anchor was secreted into the medium (B, 3rd lane). The secretion was abolished when the N-terminal signal sequence was deleted (b, 4th lane). DmGfrl isoform B, which harbors a different signal sequence than DmGfrlA, behaved similarly in these experiments (data not shown). (C) Digestion of intact DmGfrlA-expressing S2 cells with phosphatidylinositol-specific phospholipase C (PI-PLC). No DmGfrl is detected in the soluble extracellular fraction after incubation without PI-PLC (C, 2nd lane). Upon digestion with PI-PLC, a fraction of DmGfrlA was released into the soluble extracellular fraction (C, 4th lane). Only the ∼170 kDa DmGfrl species (upper band) was released, indicating that it is the mature form exposed on cell surface. (D) Heparin binding properties of DmGfrl. Immunoblot analysis of heparin bound fractions showed that DmGfrl elutes between 0.3 and 0.8M NaCl with a peak at 0.5M NaCl (upper panel). Thus, it showed lower affinity for heparin than mammalian GFRα1, which has multiple heparin binding sites and elutes at above 1.0M NaCl [24]. Similarly to the protein expressed in S2 cells the majority of V5-DmGfrlA from transgenic adult flies eluted between 0.4 and 0.7M NaCl (lower panels). (E–H) Immunofluorescence analysis of the plasma membrane localization of DmGfrlA. In detergent-permeabilized S2 cells, wild-type DmGfrlA is detected in the periphery of the cells as well as in endoplasmic reticulum and Golgi-like structures (E). In non-permeabilized cells, intracellular staining is absent but the peripheral staining detectable, indicating that DmGfrlA is exposed on the cell suface (F). In cell expressing GPI-deleted DmGfrlA, the peripheral plasma membrane staining is absent (G, H), indicating that wild-type DmGfrlA is GPI anchored on the plasma membrane. Original magnification was 600X.

In order to detect the DmGfrl protein a polyclonal antibody against a fragment containing amino acids 649–958 of the DmGfrlA isoform was raised in rabbits. Immunoblot analysis of lysates prepared from wild-type embryos, larvae, pupae and adult flies failed to detect a specific band of the predicted size, which suggest that the level of the protein was below detection limit (data not shown). However, as the protein contains a predicted N-glycosylation site and several mucin-type O-glycosylation sites we tested if we can enrich the protein by means of lectin precipitation. After testing several lectins we found that in fractions enriched with jacalin and peanut agglutinin (PNA) beads, lectins that bind mucin-type O-glycosylated proteins [22], a double band of ∼150–170-kDa was detected in adult wild-type (Oregon) flies (Fig. 4A). Given the unknown contribution of glycosylation to the molecular weight of the receptor *in vivo* it is unlikely that the different isoforms would be resolved from each other on SDS-PAGE. The ∼150–170-kDa double band was undetectable in homozygous *DmGfrl* genetrap flies (Fig. 4A, *PBac/PBac*) that showed a ∼90% reduction in *DmGfrl* mRNA level (our unpublished data), but present in heterozygous *DmGfrl* genetrap flies (Fig. 4A, *PBac/Tm3*). In transgenic *UAS-DmGfrlA* flies a protein species of similar molecular weight was detected (Fig 4A, da-Gal4xTg). The protein expressed from the transgene was also detectable with a V5 tag antibody (data not shown). Thus, we conclude that endogenous DmGfrl protein is detectable in fly tissues and it is of similar apparent molecular weight as the protein expressed from the cloned cDNA sequence.

In order to investigate the secretion and subcellular localization of DmGfrlA we ectopically expressed the protein in S2 cells. For this purpose, a V5 tag sequence was inserted after the predicted N-terminal signal sequence of DmGfrlA in the expression constructs. Immunoblotting analysis of Triton X-100 lysates from transfected S2 cells revealed two bands of ∼140 kDa and ∼170 kDa (Fig. 4B, first lane). A similar pattern of two bands was detected when the native N-terminus of DmGfrl was replaced with BiP signal sequence (Fig. 4B, 2nd lane). The apparent molecular weight of the protein expressed in S2 cells was thus in line with the molecular weight of the endogenous species detected in fly tissues. Next, mutant forms lacking the putative N-terminal signal sequence and/or the putative C-terminal GPI anchoring sequence were expressed in S2 cells (Fig. 4B). Immunoblot analysis of cell lysates and culture medium revealed that the wild-type protein was detected exclusively in the cellular fraction (Fig. 4B, first lane), whereas a mutant protein lacking the extreme C-terminus was secreted into the medium (Fig. 4B, 3 rd lane). The secretion was abolished when the N-terminal hybrophobic region was deleted, indicating that this sequence functions as a *bona fide* signal sequence (Fig. 4B, 4th lane).

To assess whether DmGfrlA is modified by a glycosylphosphatidylinositol (GPI) anchor similarly to mammalian GFRα receptors, live DmGfrA-expressing S2 cells were digested with PI-PLC (Fig. 4C). A fraction of the 170 kDa form of DmGfrlA was released into the supernatant upon PI-PLC digestion (Fig. 4C, 4^th^ lane), indicating that the protein is both exposed on the cell surface and GPI-anchored. Moreover, given that we predicted a heparin binding site on DmGfrl (see Fig. 3A) and given the role of heparin binding properties in GDNF-GFRα1-RET signaling [23], [24] we wanted to investigate whether heparin binding is conserved in insect DmGfrl. We found that a secreted form of DmGfrl in which the GPI anchoring sequence has been deleted indeed bound heparin and eluted between 0.3 and 0.8 M NaCl with a peak at ∼0.5 M NaCl (Fig. 4D). We performed a similar heparin-binding assay from adult flies overexpressing V5-DmGfrlA (Fig. 4D). Immunoblotting of the fractions revealed that the majority of V5-DmGfrlA eluted at 0.4 M to 0.7 M NaCl. Taken together, these data indicate that DmGfrl is a heparin binding protein similarly to mammalian GFRα1 receptors (24).

Finally, the subcellular localization and secretion of ectopic DmGfrlA was investigated by means of fluorescence microscopy. Immunofluorescence staining of cells expressing wild-type V5-tagged DmGfrlA revealed plasma membrane and endoplasmic reticulum (ER)/Golgi-like staining in permeabilized cells (Fig. 4E), but only plasma membrane staining in nonpermabilized cells (Fig. 4F). In cells expressing the GPI-deleted mutant protein, ER/Golgi-like staining, but no plasma membrane staining, was detected in permeabilized cells (Fig. 4G), whereas no staining was detected in non-permeablized cells (Fig. 4H). These data further support the conclusion that DmGfrlA is plasma membrane-associated and exposed on the cell surface.

### DmGfrl is Expressed in Neurons in the Embryonic Central and Peripheral Nervous Systems

**Figure 5:**
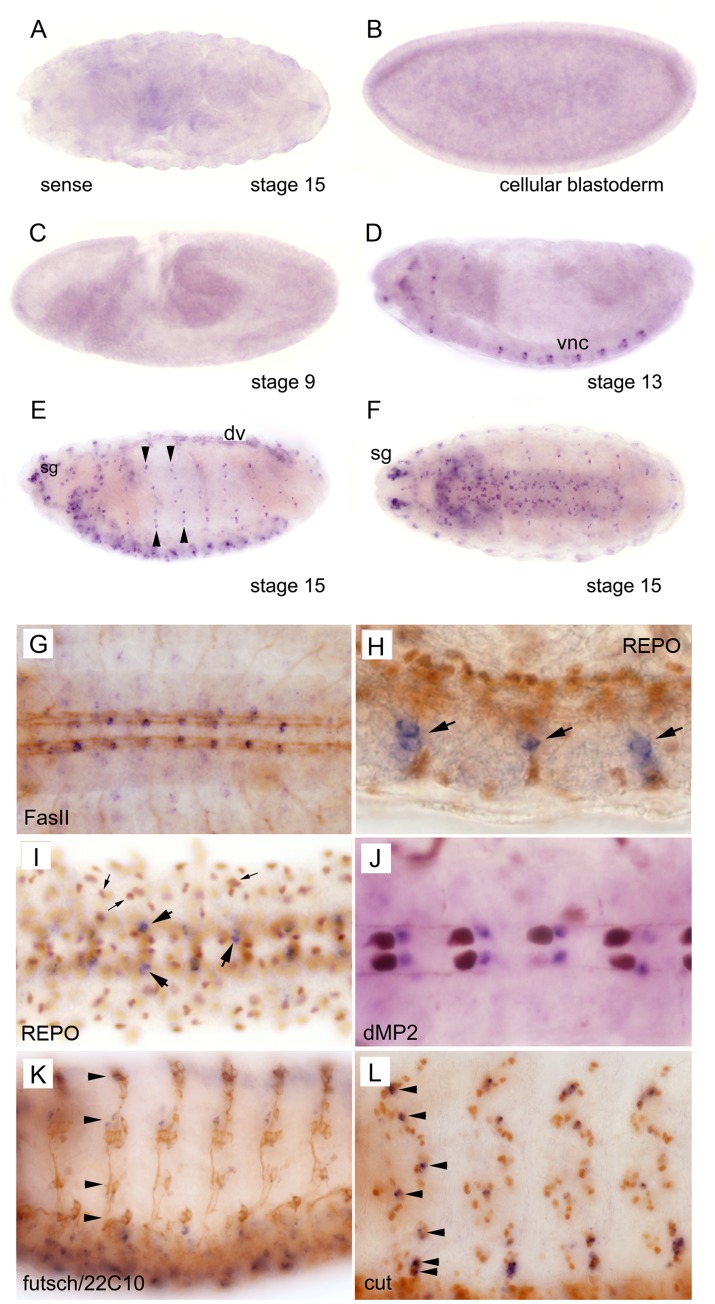
*In situ* hybridization analysis of *DmGfrl* expression during embryogenesis. Cellular blastoderm (B) and early stage embryos up to stage 10 (C) do not express *DmGfrl*. Expression (blue staining) first appeared in the seven abdominal segments in the ventral nerve cord (D, vnc) and in unidentified ganglia in the head region at about stage 13. The expression became more widespread in the central and peripheral nervous system through the later stages of embryogenesis (E, F). *DmGfrl* was expressed in single cells or clusters of a few cells in the head sensory ganglia (E, sg), ventral nerve chord (D–F) and lateral sensory ganglia (E, arrowheads). At stage 15, dorsal vessel (dv) also expressed *DmGfrl* (E). Control hybridization with a sense probe did not show specific staining (A). (G–L) Following whole mount in situ hybridization, the embryos were subjected to immunoperoxidase staining (brown color) with neuronal and glial marker antibodies. Co-staining for the neuronal marker FasII showed that *DmGfrl*-expressing cells are localized within the VNC, along the longitudinal axon bundles (G). A lateral view on the VNC shows that the *DmGfrl* signal did not colocalize with the nuclear staining for REPO, a glial cell marker (H, arrowheads show *DmGfrl*+ cells). A ventral view of the VNC also shows the paired *DmGfrl*+ cells in each segment do not co-localize with REPO (I, large arrows show *DmGfrl*+ cells). However, in the more lateral and dorsal cells there may be some overlap in the signals for DmGfrl and REPO (I, small arrows). The *DmGfrl*+ cells localized posteriorly from the dMP2 interneurons (J, brown staining) in late-stage embryos, indicating that they are not dMP2 neurons and likely not vMP2 neurons either. Futsch/22C10 staining visualizes that the lateral axonal projections along which the *DmGfrl*+ cells were located (K, arrows). Staining for cut, a sensory neuron marker showed that the *DmGfrl*-expressing cells were within the external sensory organ cell clusters and likely all positive for cut (L, arrows). Original magnification was 400X in h, i and l, and 200X in all other images.

We next investigated the expression of *DmGfrl* in *Drosophila* embryos and adult brains by means of *in situ* hybridization (Fig. 5A–F). We found that early stage *Drosophila* embryos up to stage 11 did not express *DmGfrl*, and no maternal contribution was evident (Fig. 5B, C). Expression first appeared in the seven abdominal segments in the ventral neuroectoderm at about stage 13 (Fig. 5D) where *DmGfrl* was expressed in a segmented pattern in distinct punctae of single or a few cells located laterally on both sides of the midline. At later stages expression was detected in all segments of the ventral nerve cord (VNC) and in single cells or cell clusters located more laterally from the VNC, as well as throughout the peripheral nervous system (PNS), including the head sensory complexes (Fig. 5D, E, F). Signal was also detected in unidentified cell clusters close to the oesophagus at stage 13 (Fig. 5D) and in the dorsal vessel 15 (Fig. 5E). Hybridization with a sense probe did not show specific staining (Fig. 5A).

In order to investigate the localization and identity of the *DmGfrl*-expressing cells in the embryos, the samples subjected to *in situ* hybridization were subsequently immunostained using neuronal and glial cell marker antibodies (Fig. 5G–L). In late stage embryos, the *DmGfrl*-positive cell somas were found to localize along the longitudinal FasII-positive axon bundles of the VNC (Fig. 5G). *DmGfrl*-expressing cells were negative for REPO, a glial marker in the VNC (Fig. 5H, I) suggesting that they are neurons. In an attempt to elaborate the identity of the *DmGfrl*+ cells in the VNC a co-staining for the dMP2 interneuron was performed. The paired *DmGfrl*+ cells localized posteriorly from the dMP2 neurons in late-stage embryos (Fig. 5J), indicating that they are not dMP2 neurons and likely not vMP2 neurons either, as these cells are localized anteriorly from the dMP2 neurons. On the basis of their positions, the *DmGfrl*-expressing neurons could be either aCC motoneurons or pCC interneurons, but this awaits further investigation. In the PNS, Futsch/22C10 staining showed that the lateral *DmGfrl*+ cells were located along the axonal projections of the sensory nervous system (Fig. 5K, arrows). Staining for cut, a sensory neuron marker showed that the *DmGfrl*-expressing cells were within the external sensory organ precursor cell clusters (Fig. 5L).

### DmGfrl and DmRet are Expressed in Different Cell Populations

**Figure 6:**
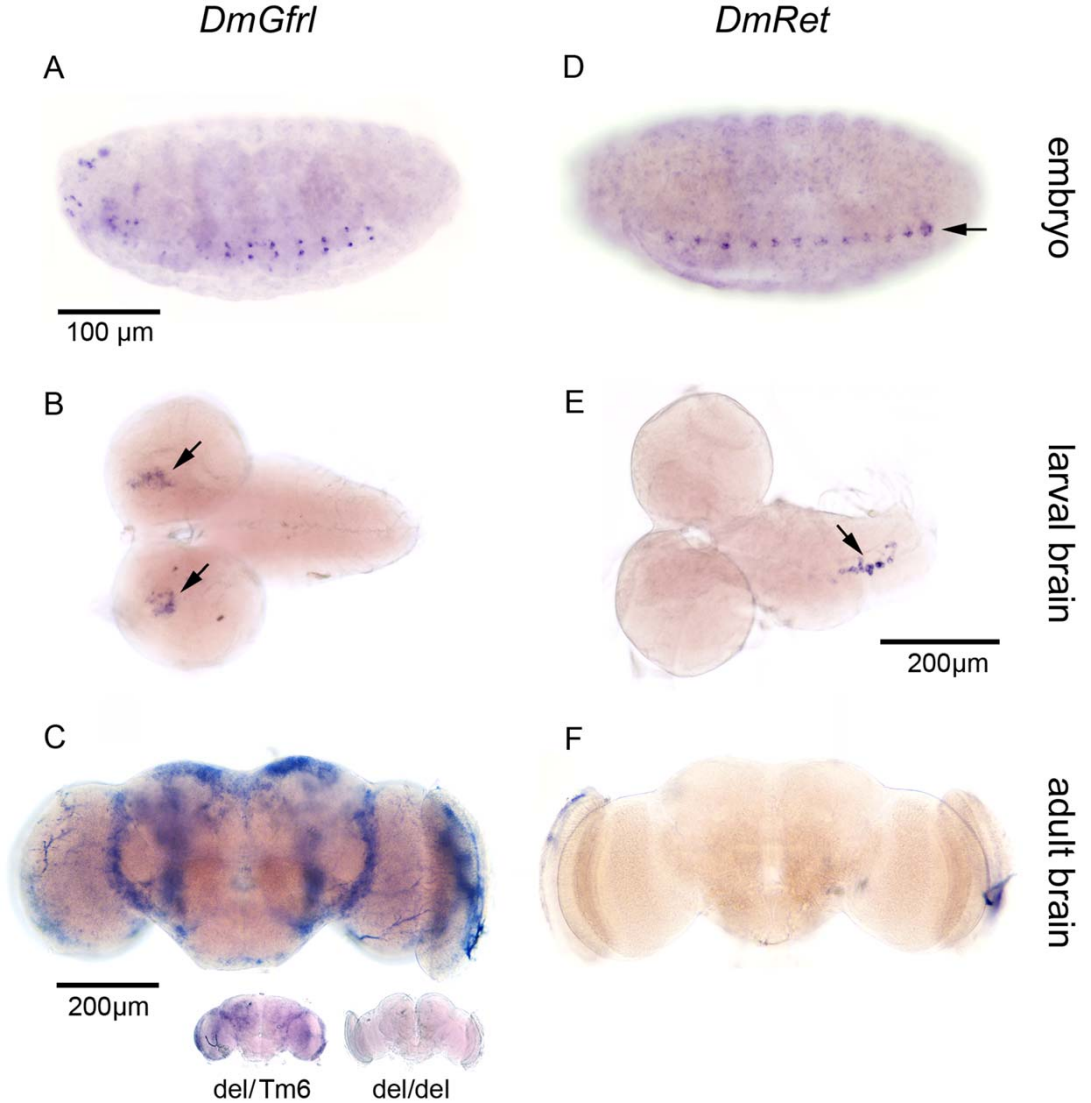
Comparison of *DmGfrl* and *DmRet* expression patterns in the *Drosophila* central nervous system throughout the development. (A–F) In the embryonic VNC at stage 13, *DmGfrl* was expressed in two rows of punctae on both sides of the midline in the seven abdominal segments (A), whereas *DmRet* was expressed at the midline in all segments (D). In 3rd instar larval brains hybridization for *DmGfrl* showed expression in two cell clusters in the dorso-medial protocerebri (B, arrows), whereas *DmRet* was expressed in a small cell population close to the midline of the ventral nerve cord (E, arrow). In the adult brain *DmGfrl* mRNA was widely distributed (C) whereas *DmRet* was absent or expressed at a level below detection limit (F). Homozygous *DmGfrl* mutant adult brain did not show any signal in in situ hybridization (C, inset, *del/del*), whereas a heterozygous mutant brain did show a pattern similar to wild-type brain (C, inset, *del/Tm6*). The scale bars indicate the scale of each image, except for the inset in image C.

Surprisingly, we did not detect *DmGfrl* expression in several embryonic tissues in which *DmRet* expression has been previously reported [10], including the yolk sac, stomatogastric nervous system anlage and the Malpigian tubule anlage. In the ventral nerve cord, *DmRet* (Fig. 6D) was expressed in a segmented, punctate pattern, and in contrast to *DmGfrl* (Fig. 6A), at the midline. *In situ* hybridization analysis of *DmGfrl* and *DmRet* expression in later developmental stages revealed that *DmGfrl* expression continues in the central nervous system in larval and adult stages (Fig. 6B, C). In 3rd instar larval brains, *DmGfrl* expression was detected in symmetrically located foci in the posterior part of the brain lobes (Fig. 6B). In contrast, *DmRet* expression was detected in a small cell population located along the midline of the larval ventral nerve cord (Fig. 6E). This expression pattern is in line with published data, in which *DmRet* mRNA was detected in a putative neurosecretory cell cluster in the larval VNC [25]. In the adult fly brain *DmGfrl* mRNA was abundant and mainly detected in the cell somas located in the central brain in both female and male flies (Fig. 6C). Interestingly, this resembles the expression pattern of GABA_B_ receptor 2 that is expressed in GABAergic interneurons in the adult fly brain [26]. In contrast, *in situ* hybridisation with different *DmRet* probes did not give any detectable expression in the adult brain (Fig. 6 F). Taken together, we found that *DmGfrl* and *DmRet* are expressed in a strikingly non-overlapping pattern throughout the development of the fly nervous system.

### *DmGfrl* is Required for Egg Morphogenesis

**Figure 7:**
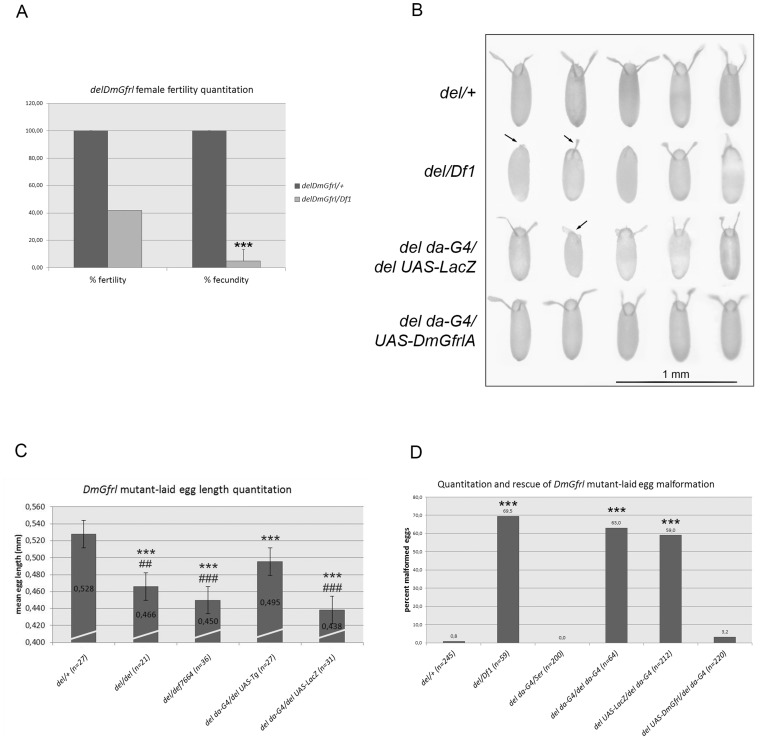
Quantitation of *DmGfrl* null female fertility and oogenesis phenotype. (A) *delDmGfrl* females displayed markedly reduced fertility. The absolute fertility of *delDmGfrl/Df1* transheterozygous females was reduced by ∼60% as compared to heterozygous control females. Their fecundity (measured as the average number of progeny produced by individual females in a given time) was reduced to ∼10% of the fecundity of control females. (B) Morphology of eggs laid by control, *delDmGfrl* and rescue females. Uppermost row shows the morphology of heterozygous (*delDemGfl/+*) control eggs. Second row exemplifies the morphology of eggs laid by *DmGfrl* null females (*delDmGfrl/Df1*). The eggs were small and often translucent (not visible in this image), and ∼60% of them display lack of or abnormal dorsal appendages (arrows). *LacZ* transgene under the *daughterless* (*da)* driver (3^rd^ row) did not rescue the egg morphology, whereas eggs laid by females expressing the *DmGfrl* transgene under the *da* driver in *DmGfrl* null background (4^th^ row) were almost fully wild-type by appearance. (C) Quantitation of the size of eggs laid by control, *delDmGfrl* and rescue females. Average egg length was reduced from 0.528 mm in heterozygous control eggs (female genotype *delDmGfrl/+)* to 0.466 mm in eggs laid by homozygous *delDmGfrl*/*delDmGfrl* females and to 0.450 mm in eggs laid by *delDmGfrl/Df1* females (1^st^ to 3^rd^ bars). *DmGfrl* transgene (UAS-Tg), but not *LacZ* transgene (UAS-LacZ), partially rescued the dumpless-like phenotype (4^th^ and 5^th^ bars). Statistical significance from Tukey’s post hoc test after one-way ANOVA are shown with asterisk (*) with respect to the *del/+* genotype and hash (#) with respect to the transgene rescue genotype (*del da-G4/del UAS-DmGfrl*). (D) Quantitation and rescue of the malformed egg phenotype. The percentage of malformed eggs laid by *DmGfrl* null females, displaying either dumpless-like phenotype or malformed dorsal appendages or both, was ∼60–70% depending on the genetic background (2^nd^, 4^th^ and 5^th^ bars). Expression of DmGfrl under the *da-GAL4* driver diminished the percentage of malformed eggs from 63% (driver only, 4^th^ bar) to ∼3% (driver and transgene, 6^th^ bar). Expression of *LacZ* transgene in the same background did not rescue the egg phenotype (59%, 5^th^ bar). Asterisk (*) represent statistical significance obtained from Dunn’s post hoc test after non-parametric Kruskal-Wallis ANOVA with respect to the *del/+* genotype. The error bars represent standard deviation in graphs A, C, and D. Two asterisks correspond to p-values of <0.01 and three asterisk to p-values of <0.001.

The *DmGfrl* locus does not harbor a P element suitable for the generation of a loss-of-function allele by the traditional imprecise excision strategy. However, we generated a *DmGfrl* null allele (referred to hereafter as *delDmGfrl* or *del*) by FLP-mediated genomic deletion between two FRT-containing PBac elements (see Fig. 1). Genomic PCR demonstrated loss of the *DmGfrl* genomic region and RT-PCR loss of *DmGfrl* mRNA in homozygous flies (Fig. S3). Adults homozygous for the deletion allele were viable and apparently normal (data not shown). However, we could not establish a homozygous stock, which suggested that loss of *DmGfrl* affects the reproduction of the flies. To assess the fertility of the mutant females we first crossed *delDmGfrl* flies with flies carrying a genomic deficiency (*Df1*) in the *DmGfrl* region. We then mated the resulting *del/Df1* females and heterozygous (*del/+*) control females with wild-type males and scored the progeny (Fig. 7A). Heterozygous *del/+* females (Fig. 7A) displayed fertility comparable to wild-type flies (data not shown). *del/Df1* females displayed a reduction of ∼60% in absolute fertility and ∼90% in fecundity (e.g. number of progeny per female at 72 hours post-mating) as compared to *del/+* females (Fig. 7A). This drastic reduction in fecundity suggests that *DmGfrl* null flies have a defect in oogenesis. In line with this interpretation, we observed that *del/del* and *del/Df* females laid very few eggs. Further inspection showed that a large fraction of eggs laid by mutant females were small and translucent, reminiscent of dumpless phenotype, and did not develop into larvae. We performed a rescue experiment with the *DmGfrl* transgene and imaged the appearance of eggs laid by females of the control, mutant and rescue genotypes (Fig. 7B). Control genotype (*del/+*) showed normal egg appearance with normally formed dorsal appendages, whereas mutant genotype (*del/Df1)* showed small size and lacking or malformed dorsal appendages (Fig. 7B, arrows). The egg morphology defect was efficiently rescued by the *DmGfrl* transgene (*del UAS-DmGfrl/del da-G4*) but not by a *LacZ* transgene driven by *da* driver in the mutant background *(del UAS-LacZ/del da-G4*) (Fig. 7B). We quantified the dumpless-like phenotype by measuring the length of eggs laid by mutant and rescue females. The egg length was reduced in mutant genotypes (*del/Df1, del/del, del da-G4/del UAS-LacZ*) and the difference to heterozygous control (*del/+*) genotype was statistically highly significant between all genotypes (Fig. 7C, one-way ANOVA with Tukey’s posthoc test). More importantly, the reduction in egg length was partially rescued by the *DmGfrl* transgene but not by the *LacZ* transgene in statistically highly significant manner (Fig. 7C). The morphogenesis defect in eggs laid by *DmGfrl* mutant and rescue females was further quantified on apple juice plates after 20 hours of egg laying, and the data is presented in Fig. 7D. The morphology defect was statistically highly significant between mutant genotypes and the control genotype (Kruskal-Wallis non parametric test p<0.0001, comparisons between groups are shown with asterisks by Dunn’s post hoc test). About 60–70% of eggs laid by the mutant females were malformed, depending on the genetic background (*del/Df1, del da-G4/del da-G4, del UAS-LacZ/del da-GAL4*) as opposed to only 0.8% in heterozygous del*DmGfrl* background (*del/+*). Expression of the *DmGfrl* transgene under the *daughterless* (*da*) driver (*del UAS-DmGfrl/del da-GAL4*) rescued the egg morphology so that the percentage of malformed eggs diminished from ∼60% to 3.2%, whereas a *LacZ* transgene did not rescue the defect (*del UAS-LacZ/del da-GAL4*). Similar rescue was observed when using the strong *actin* driver (data not shown). Taken together, these results indicate that the *Drosophila* Gfr-like receptor regulates oogenesis, a function previously unrecognized in mammals.

### DmGfrl and FasII Interact Genetically to Control Male Fertility

**Figure 8:**
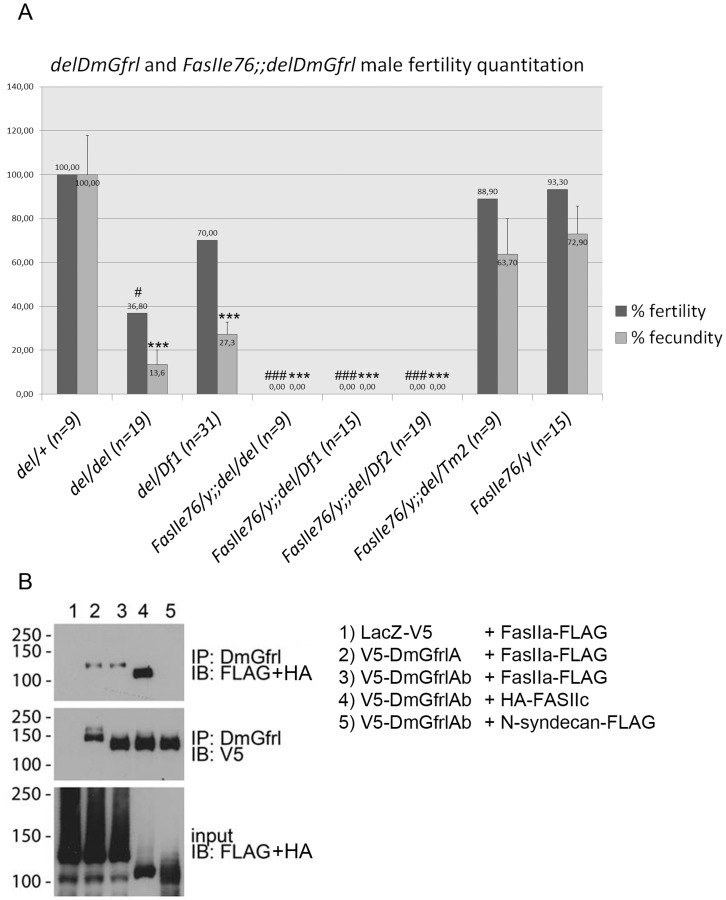
Quantitation of *DmGfrl* mutant male fertility and evidence for fertility-controlling *DmGfrl-FasII* genetic interaction. (A) *delDmGfrl* and *delDmGfrl/Df1* males display reduced fertility (37% and 70% of *del/+* control, respectively) and fecundity (14% and 27% of *del/+* control, respectively). Males carrying the *FasII^e76^* allele alone or together with one copy of the Δ*DmGfrl* allele (*FasII^e76^;;del/Tm2*) displayed only mildly reduced fertility (89% and 93%, respectively) and fecundity (64% and 73%, respectively). In contrast, males carrying the hypomorphic *FasII^e76^* allele, and homozygous for the *delDmGfrl* allele or compound heterozygous for *delDmGfrl* allele and either of two different a genomic deficiencies (*Df1* or *Df2*, see Experimental procedures), never produced progeny (fertility and fecundity 0%). Oneway ANOVA with Bonferroni’s post hoc test was used for statistical analysis of the data. Asteriks (*) and hash (#) represent statistical significance with respect to the control phenotype (*del/+*). One asterisk corresponds to p-values of <0.05 and three asterisks to p-values of <0.001. (B) Biochemical interaction of DmGfrl and FasII receptors. Ectopic FasIIa, the transmembrane isoform of the FasII receptor, coimmunoprecipitated with both DmGfrlA and DmGfrlAb from S2 cell lysates (B, uppermost panel, 2^nd^ and 3^rd^ lanes). Interestingly, also the GPI-anchored FasII isoform FasIIc coimmunoprecipitated with DmGfrl (B, uppermost panel, 4^th^ lane). In contrast, *Drosophila* N-syndecan did not coprecipitate with DmGfrl (B, uppermost panel, 5^th^ lane). No coprecipitation of FasII was seen with LacZ (B, uppermost panel, 1^st^ lane). The middle panel verifies the presence of the bait (V5-DmGfrl) in the immunoprecipitates and the lowermost panel the presence of the prey proteins in the input lysates. Molecular weight markers (in kDa) are shown on the left-hand side of the panels.

Given that GDNF and its receptors are known to be required for spermatogenesis in mammals [27], we wanted to assess the fertility of *DmGfrl* mutant males. To this end, the fertility and fecundity of the mutant males was quantified essentially as described above. We found that homozygous *del/del* males and *del/Df1* transheterozygous males displayed statistically significally reduced fertility (Fig. 8A, Kurskal-Wallis non-parametric ANOVA, p<0.0001, comparisons between the genotypes by Dunn’s post hoc test are shown with asterisks) and fecundity (Fig. 8A, one-way ANOVA F(7,112) = 17.05, p<0.0001, results from Bonferroni’s post hoc test are shown with asterisks). We had no suitable hypomorphic *DmRet* allele to investigate possible genetic interaction between *DmGfrl* and *DmRet* in the context of either male or female fertility. However, we tested whether a genomic *DmRet* deficiency (*DmRetDf*) could modify the fertility phenotype of *DmGfrl* mutant males. Not unexpectedly, *DmRet* heterozygosity had no significant effect on the fertility defect caused by the *delDmGfrl* allele (Fig. S5).

As mammalian GFRα1 and NCAM receptors are known to interact and mediate RET-independent GDNF signaling in the nervous system [8], we utilized the *delDmGfrl* allele to look for a genetic interaction with the *Drosophila* NCAM homolog FasII. We combined *FasII^e76^*, a well-characterized hypomorphic allele with ∼10% residual protein expression [28], with the *delDmGfrl* allele. We crossed this stock with two different *DmGfrl* deficiency lines (*Df1*, *Df2*) or an independent deletion allele and scored the number of eclosing males. Double hemizygous/transhomozygous adult males appeared at a normal Mendelian ratio and were grossly normal and viable (data not shown). We then went on to assess if reduced FasII levels in this stock could modify the male fertility phenotype we observed in *DmGfrl* mutant flies. We found that *FasII^e76^;;del/del* and *FasII^e76^;;del/Df1* males were completely infertile in this assay. In fact, despite abundant egg laying by the females mated with *FasII^e76^;;del/del* males we never observed larvae in the vials even after several weeks. As the *delDmGfrl* allele and *Df1* remove two small genes embedded in a long *DmGfrl* intron, we wanted to rule out the effect of these genes on male fertility by utilizing another genomic deficiency (*Df2*) that only removes 5′ exons of *DmGfrl* but leaves these two genes intact. Again, males with this genotype (*FasII^e76^;;del/Df2)* were completely infertile (fertility and fecundity 0%, Fig. 8A). Homozygous *FasII^e76^* males displayed fertility and fecundity close to those observed in wild-type control flies (Fig. 8A). These data indicate that *DmGfrl* and the *FasII^e76^* allele interact genetically to control male fertility.

Finally, to investigate whether DmGfrl and FasII receptors can interact at the protein level, we expressed the tagged receptors in S2 cells and performed coimmunoprecipitation experiments (Fig. 8B). We found that both FasIIa and FasIIc isoforms coprecipitated with DmGfrlA and DmGfrlAb isoforms, but not with the control transmembrane protein N-syndecan (Fig. 8B). This biochemical interaction, though not conclusive evidence for physical interaction, suggests that a physical interaction *in vivo* may underlie the genetic interaction we observed between *DmGfrl* and *FasII^e76^*.

## Discussion

At the start of this project, two *Drosophila melanogaster* cDNA fragments predicting amino acid sequence with similarity to the GFRα domains of mammalian GDNF receptor proteins had been annotated in the Genbank [9]. Starting from these cDNA fragments, we used RACE, RT-PCR and *in silico* sequence analysis to assemble what we presume is the full genomic structure of the gene, and identified altogether six transcripts produced from this locus. Based on previously suggested nomenclature [9], we named this gene *Drosophila melanogaster Glial cell line-derived neurotrophic factor family receptor-like*, or *DmGfrl*. The two major *DmGfrl* transcripts (A and B) detectable on Northern blots were found to differ only in their 5′ untranslated regions and the 5′ coding sequence preceding the first GFRα-like domain, including the translation initiation site and a predicted signal sequence. The exons harboring the translation start sites for transcript A and B are separated in the genome by ∼27 kb, which indicates that the two main transcripts are very likely to have separate promoter regions. Such differential promoter usage may serve to allow regulation of the same gene product by separate sets of transcription factors in different developmental and/or physiological contexts [29]. Indeed, *DmGfrl* transcript A is predominant in embryos. Both major *DmGfrl* transcripts encode a protein with four cysteine-rich GFRα-like domains, which is in line with previous *in silico* predictions [9], [30]. Similarity to the mammalian GFRα receptors is restricted to these domains, which have a characteristic arrangement of 10 cysteine residues in each domain (see Fig. S1). Interestingly, a *Gfr-like* gene in *C. elegans* predicts a similarly large protein of >1000 amino acids with four GFRα-like domains. Based on gene structures a common origin has been proposed for the exons encoding D1 to D3 in insect and sea urchin Gfr-like proteins and vertebrate GFRα genes, which suggests that a protoGFRα receptor evolved before the protostome-deuterostome divergence [30].

Insects lack GDNF family ligands, but having cloned the *Drosophila* receptor homologs we asked whether they might respond to mammalian GDNF and whether DmGfrl could mediate mammalian RET phosphorylation. Both experiments suggested that DmRet and DmGfrl are not structurally sufficiently conserved to bind to mammalian GDNF or interact with the mammalian receptor homologs (Fig. S2). It is interesting to speculate that one of the seven *Drosophila* TGF-β ligands could function as a soluble ligand (“protoGDNF”) for DmRet and/or DmGfrl.

During *Drosophila* embryogenesis, *DmRet* is expressed in many tissues that are functionally analogous to those in which mammalian *RET* is expressed, including foregut neurons, the excretory system, peripheral ganglia and the central nervous system [10]. We compared *DmGfrl* and *DmRet* expression in the embryonic nervous system and in the larval and adult brain using in situ hybridization. The expression pattern of *DmGfrl* was generally concordant with the neuronal cell expression of *GFRα1* and *GFRα2* in mice, in which expression at both the mRNAs and proteins has been reported in several brain areas, the spinal cord and various peripheral ganglia [31], [32]. Interestingly, however, we did not detect *DmGfrl* expression in the Malpighian tubules, the *Drosophila* analog of mammalian kidney. In line with previously published *in situ* hybridization data [10], [11] we found that *DmRet* was first expressed in the yolk sac (data not shown), and subsequently in the ventral neuroectoderm starting from embryonic stage 13. *DmRet* and *DmGfrl* expression coincided temporally but not spatially during embryogenesis. In the larval and adult brain, *DmGfrl* and *DmRet* expression patterns were also completely non-overlapping. Thus, we conclude that DmRet and DmGfrl likely do not function as an *in cis* receptor-co-receptor pair as do mammalian RET and GFRα receptors. However, our data do not rule out the possibility that DmRet and DmGfrl could interact via an alternative mode, for example *in trans* (cell-to-cell) or by cleavage and diffusion of soluble DmGfrl. In the absence of a *DmRet* null allele or a suitable hypomorphic allele we looked for a genetic interaction between *DmRet* and *DmGfrl* in misexpression experiments (Fig. S4). We did not find evidence that DmGfrl coexpression could modify ectopic DmRet-induced phenotype in the eye (Fig. S4). The ectopic expression experiment is, however, inconclusive, and progress in this direction will require the generation of a *DmRet* allele suitable for genetic interaction experiments.

To gain insight into the *in vivo* function of the DmGfrl receptor we generated a *DmGfrl* null allele by FLP-mediated genomic deletion. *DmGfrl* null flies were grossly normal and viable. However, they displayed a severe defect in both male and female fertility. The reduced female fertility results from an oogenesis defect as the mutant females laid fewer eggs than normally and a large fraction of those were small and had abnormal dorsal appendages. The egg morphology defect was efficiently rescued by transgene expression under the widely active *daughterless* and *actin* drivers, indicating that the phenotype is specific to loss of *DmGfrl* expression, and likely dependent on the somatic tissue of the ovary. However, the transgene did not rescue the reduced viability of the eggs or the reduced fecundity of the females (data not shown). This suggests that the reduced egg viability is either dependent on germline cells, in which this transgene should not be expressed [33], or is not rescued by the DmGfrlA isoform used in these experiments. Similarly to females, we observed in *DmGfrl* null males a fertility defect that was not fully penetrant. Because the fecundity of *DmGfrl* null males was much more reduced than their absolute fertility, we reason that a defect in spermatogenesis is a likely cause. Dissection of the testis histology and function in the mutant flies, as well as further rescue experiments will likely clarify the mechanism of the fertility defect in *DmGfrl* null males. Interestingly, on the basis of a proteomics study DmRet protein is present in adult spermatozoa [34], which warrants studies of the putative conserved function of DmRet in spermatogenesis [27].

Finally, on the basis of molecular evidence from mammals we wanted to look if DmGfrl might interact with the *Drosophila* NCAM homolog FasII. In mammals NCAM binds GDNF and GFRα1 and functions as an alternative signaling receptor for GDNF, mediating neuronal migration and axonal growth [8]. FasII is widely expressed in the embryonic VNC [28], making it likely that it is also expressed in the *DmGfrl*-expressing neurons. We combined a hypomorphic *FasII* allele with the *delDmGfrl* allele and investigated if the former could modify the male fertility phenotype of *DmGfrl* null flies. Strikingly, the double homozygous males were completely infertile, indicating a strong genetic interaction between *DmGfrl* and the *FasII* allele. There is currently little data linking NCAM/FasII function to reproduction. Nevertheless, on the basis of *in silo* data both *DmGfrl* (see Flybase FBgn0262869) and *FasII* (see Flybase FBgn0000635) are expressed at low levels in the testis and ovary. There is evidence for a role of FasII in the hormonal control of the development of *Drosophila* male genitalia, as a *FasII^spin^* allele has been shown to disrupt the looping of the male genitalia and spermiduct [35]. Interestingly, in *FasII^spin^* flies, the innervation of corpora allata in the ring gland is disrupted, which the authors suggest may lead to elevated level of juvenile hormone and eventually to the looping defect [35]. In preliminary inspection, we did not observe a rotation defect in the external male genitalia in *FasII^e76^;;del/Df* males (data not shown), but this lead will be worth further study. We did not observe any gross embryonic or adult neuronal phenotypes in the *DmGfrl* null flies. As subtle developmental or behavioral phenotypes may be present this question will require careful further studies.

The strong genetic interaction between *DmGfrl* and *FasII* that we describe is corroborated by our data showing biochemical interaction between the ectopically expressed receptors. To the best of our knowledge these data are the first to suggest that the GFRα1-NCAM interaction described in mammalian systems is evolutionarily conserved. Together with our results suggesting that DmRet and DmGfrl do not function *in cis* in *Drosophila*, which lacks GDNF ligands, these data imply that DmGfrl may be an evolutionarily ancient binding partner for NCAM/FasII. Whether or not a soluble ligand exists in *Drosophila* and is needed to activate the putative FasII-DmGfrl signaling complex needs to be tackled in future studies.

## Supporting Information

Figure S1 Conservation of the cysteine pattern of the GFRα-like domains in vertebrates and invertebrates. (A) Conserved cysteine pattern in DmGfrl GFRα-like domains 0 to 3. The (partly) conserved cysteines are in red font and numbered from 1 to 10 above the alignments. (B) Alignment of the amino acid sequence of GFRα domain 2 of DmGfrl and GFRα domains from various organisms. All four GFRα domains of Gfr-like proteins are highly conserved between *Drosophila* species, as exemplified by *Drosophila virilis* (Dvir) with an amino acid identity of ∼98% and similarity of ∼100%. In comparison to *Apis mellifera* these numbers are approximately 74% and 88%, to *Caenorhabditis elegans* 24% and 45%, *Gallus gallus* 30% and 55%, *Ciona intestinalis* 29% and 43% and to *Rattus norvegicus* 28% and 44% (Gfrαl). “pp” denotes predicted protein.
(PDF)

Figure S2 Assay for responsiveness of *Drosophila* Ret and Gfrl to mammalian GDNF. (A) DmRet phosphorylation assay. Cell lines stably transfected with DmRet-3xFLAG or DmRet-3xFLAG and V5-DmGfrl expression constructs were induced with copper sulphate for 2 hours and then stimulated with 50 ng/ml recombinant human GDNF for 1 hour. As an expression level control, a sample from cells induced for 24 hours was loaded (upper and lower panel, 1^st^ lanes). In the cell line expressing DmRet only, addition of GDNF did not induce DmRet tyrosine phosphorylation above the basal level (upper panel, 2^nd^ and 3^rd^ lanes). In the cell line expressing DmRet and DmGfrl, GDNF did not cause a discernible increase in DmRet phosphorylation upon GDNF addition either (upper panel, 4^th^ and 5^th^ lanes), indicating that human GDNF does not stimulate DmRet with or without DmGfrl. Equal loading of DmRet was verified by reprobing the filter with anti-FLAG antibody (lower panel). (B) Mammalian RET phosphorylation assay. MG87RET cells were transfected with plasmids encoding GFRα1, V5-DmGfrlA or V5-DmGfrlB. The cells were serum-starved and then stimulated with 50 ng/ml recombinant human GDNF. RET tyrosine phosphorylation was assayed from RET immunoprecipitates with anti-phosphotyrosine antibody (B, upper panel). GDNF induced RET phosphorylation above background level only in cells transfected with GFRα1 (3^rd^ lane) but not in cells transfected with V5-DmGfrlA or V5-DmGfrlAb (4^th^ and 5^th^ lanes). Equal loading of RET was verified by probing the blot with RET antibody (B, lower panel). Molecular weight markers are shown on the left side of the panels.
(PDF)

Figure S3 Genotyping and verification of loss of *DmGfrl* expression in FLP deletion flies. (A) Genomic PCR with primers specific for the *PBac* elements used in the deletion strategy verifies the presence of remnants of both *PBac* elements on the same chromosome, indicating correct excision (A, upper panel). Genomic DNA from homozygous and heterozygous adults from two independent deletion lines (3C and 19F) was isolated and subjected to PCR analysis. *Oregon* and *w-* were used as negative controls (1^st^ and 7^th^ lanes) and a mixture of genomic DNA from the parental *PBac* lines as a positive control (6^th^ lane). PCR with *DmGfrl* genomic primers located within the deletion (A, lower panel) verifies loss of the genomic region in homozygous flies (3^rd^ and 5^th^ lanes). (B) RT-PCR analysis verifies loss of *DmGfrl* mRNA expression, but not *DmRet* expression, in flies homozygous for the deletion alleles (2^nd^ and 4^th^ lanes). Expression is detected *w-* flies (1^st^ lane) and in flies heterozygous for the deletion alleles (3^rd^ and 5^th^ lanes) or for a genomic deficiency (6^th^ lane).
(PDF)

Figure S4 Phenotypes caused by *in vivo* DmGfrl and DmRet misexpression. (A–H) Newly eclosed adult flies (non-balanced and balanced, when applicable) from the crosses were counted and inspected. The V5-DmGfrl transgene did not cause gross developmetal lethality or apparent adult phenotype when driven with *tubulin*, *da*, *elav* (data not shown), *Ey GMR* (B) or *nubbin* (E) drivers. DmRet-FLAG driven with *tubulin* driver caused embryonic lethality (data not shown), but, surprisingly, no gross lethality with *da*, *elav* (data not shown) or *TH* (H). Driving DmRet expression with the eye-specific *Ey GMR* (C), wing-specific *Nub* (F) and tyrosine hydroxylase expressing cell-specific *TH* (H) drivers caused rough eye, malformed wing and perpendicular wing position, respectively. Co-expression of both receptors in the eye (*Ey GMR-Gal4*) did not modify the DmRet-induced rough eye phenotype (data not shown). Furthermore, co-expression of both receptors in the nervous system (*elav-Gal4*) did not cause any obvious synthetic phenotypes (data not shown).
(PDF)

Figure S5 Effect of *DmRet* deficiency on *DmGfrl* mutant male fertility. The effect on male fertility of *DmRet* genomic deficiency (*DmRetDf*) in combination with the *DmGfrl* mutant allele (*del*) was quantified as described in Materials and Methods. The fertility and fecundity of double heterozygous (*DmRetDf/+;del/+*) control males was set as 100%. The *del/del* genotype showed statistically highly significantly reduced fertility as compared to the control genotype (non-parametric ANOVA, Kruskall-Wallis with Dunn’s posthoc test, p = 0.0008). The fecundity of both the *del/del* and the *DmRetDf/+;del/del* genotypes was statistically highly significantly reduced as compared to the control genotype (one-way ANOVA with Bonferroni’s posthoc test, p<0.0001). In contrast, there was no statistically significant difference in either fertility or fecundity between the *del/del* and *DmRetDf/+;del/del* genotypes, which indicates that *DmRet* heterozygosity did not affect the fertility of DmGfrl mutant males. Error bars represent standard error of mean (SEM).
(PDF)

Table S1 Genomic locations and features of DmGfrl exons on 3R:16310273-16204718.
(PDF)
