# Supplementary material for: Characterization of Drosophila GDNF Receptor-Like and Evidence for Its Evolutionarily Conserved Interaction with Neural Cell Adhesion Molecule (NCAM)/FasII
Source: PLoS One. 2012 Dec 20;7(12):e51997. doi: 10.1371/journal.pone.0051997 (PMC3527400; doi:10.1371/journal.pone.0051997)
Supplement: Figure S1 — Conservation of the cysteine pattern of the GFRα-like domains in vertebrates and invertebrates. (A) Conserved cysteine pattern in DmGfrl GFRα-like domains 0 to 3. The (partly) conserved cysteines are in red font and numbered from 1 to 10 above the alignments. (B) Alignment of the amino acid sequence of GFRα domain 2 of DmGfrl and GFRα domains from various organisms. All four GFRα domains of Gfr-like proteins are highly conserved between Drosophila species, as exemplified by Drosophila virilis (Dvir) with an amino acid identity of ∼98% and similarity of ∼100%. In comparison to Apis mellifera these numbers are approximately 74% and 88%, to Caenorhabditis elegans 24% and 45%, Gallus gallus 30% and 55%, Ciona intestinalis 29% and 43% and to Rattus norvegicus 28% and 44% (Gfrαl). “pp” denotes predicted protein. (PDF) [file pone.0051997.s001.pdf]

A

```
                   1          2        3               4              5        6                        7 8                        9                 10
DmGfrl D0   ILNCILARQLCFEDPSCSAILEIIPRVCGPIPVSCSTV-TVTKCQAALRTLQAFQFFRPTCLCK—EPGMDPD-------CNHFRDFLFDHPCGFV
DmGfrl D1   LPTCNHALSVCQQERKCLKLFEDFKTHCKVRD-NKCKMENRDAC--HDSWTNLRLSPMFGCICPNNHMKKR--------CDRIFNIVNHNPCVVR
DmGfrl D2   QSTCHTALDTCREDPSCSSSLQPMLTHCELHRCNRNACMSSLQAFYKGPHEDLNLDIAF-CLCKKTSSQQNGNGNRHDMC-MIAQEKLHPVCAQR
DmGfrl D3   PPACHVVADSCKEDRECRLKLEYYEQACAVDSVTKKCAGRPSGC-RTAMIGILGTMLRTTCACQGTDPQHLYQ------CVGWRRLLWMNPCVVE
```

B

```
                               1          2       3              4             5             6                                       7 8                 9              10
Dmel Gfr-like D1               CNHAL-SVCQQERKCLKLFEDFKTHCKVR---DNKC--KMENRDACHDSWTNLRLSPM-------------FGCICPNN--HMKK--RCDRIFNIVNHNPC
Dvir Gfrl-like                 CNHAL-SVCQQERKCLKLFEDFKTHCKVR---DNKC--KMENRDACHDAWTNLRLSPM-------------FGCICPNN--HMKK--RCDRIFNIVNHNPC
Apis mellifera pp              CNHAL-SVCLRGKPCSQIYEDFKSNCKAR---EGKC--RMENRSACHDSWTQLRLSPM-------------FGCICPNN--HMKR--RCDRIFSTVNHNPC
C.elegans pp                   CDAALYQVCLKHVSCSQLWSMFRKNCDVDL--DNQC--RMADREVCWQSFEGLTWSGL-------------GDCQCASSNSSD-----CHWIRLHTNYNKC
Ciona intestinalis pp          CLQSL-KNCKRNSTCKEVYKSVKNKCKAK---EAQCNARTADLQQCAMDIAYLREAAFPID----------NKCICHGKSLSLKKLVRCSEILNTVYANPC
Gallus gallus Gfrα1            CTIAK-RVCQEDLYCSSVYRSFQRACRAE---AAKC--RMGSQE-CLSAWKELRKTVL-------------GECKC-SEPL-Q---RRCLRIWKGIFSNPC
R.norwegicus Gfrα1  D3         CLSLQ-DSCKTNYICRSRLADFFTNCQPESRSVSNC—LKENY-ADCLLAYSGLIGTVXTPNYVDSSSLSVAPWCDCSNSGNDLED---CLKFLNFFKDNTC
```
