# Supplementary figures and images for: Characterization of Drosophila GDNF Receptor-Like and Evidence for Its Evolutionarily Conserved Interaction with Neural Cell Adhesion Molecule (NCAM)/FasII
Source: PLoS One. 2012 Dec 20;7(12):e51997. doi: 10.1371/journal.pone.0051997 (PMC3527400; doi:10.1371/journal.pone.0051997)

A

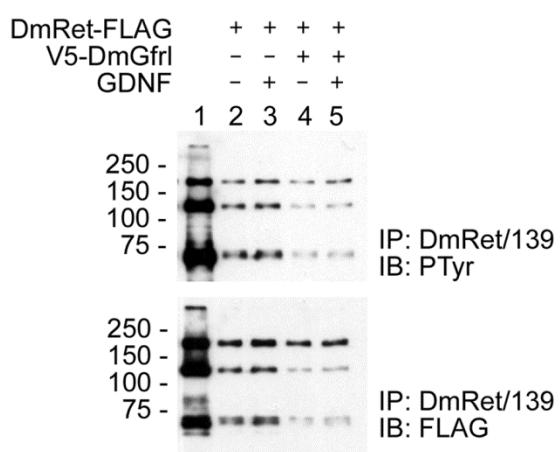

B

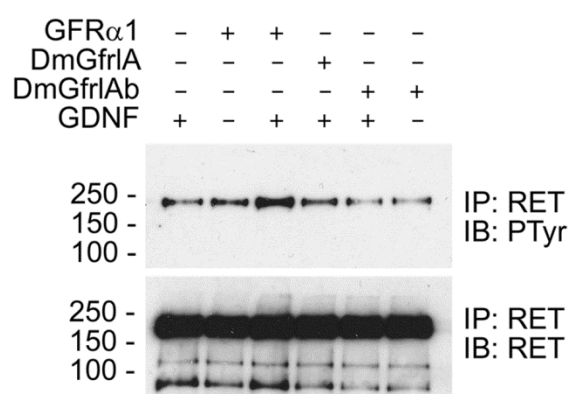

Supplement: Figure S2 — Assay for responsiveness of Drosophila Ret and Gfrl to mammalian GDNF. (A) DmRet phosphorylation assay. Cell lines stably transfected with DmRet-3xFLAG or DmRet-3xFLAG and V5-DmGfrl expression constructs were induced with copper sulphate for 2 hours and then stimulated with 50 ng/ml recombinant human GDNF for 1 hour. As an expression level control, a sample from cells induced for 24 hours was loaded (upper and lower panel, 1st lanes). In the cell line expressing DmRet only, addition of GDNF did not induce DmRet tyrosine phosphorylation above the basal level (upper panel, 2nd and 3rd lanes). In the cell line expressing DmRet and DmGfrl, GDNF did not cause a discernible increase in DmRet phosphorylation upon GDNF addition either (upper panel, 4th and 5th lanes), indicating that human GDNF does not stimulate DmRet with or without DmGfrl. Equal loading of DmRet was verified by reprobing the filter with anti-FLAG antibody (lower panel). (B) Mammalian RET phosphorylation assay. MG87RET cells were transfected with plasmids encoding GFRα1, V5-DmGfrlA or V5-DmGfrlB. The cells were serum-starved and then stimulated with 50 ng/ml recombinant human GDNF. RET tyrosine phosphorylation was assayed from RET immunoprecipitates with anti-phosphotyrosine antibody (B, upper panel). GDNF induced RET phosphorylation above background level only in cells transfected with GFRα1 (3rd lane) but not in cells transfected with V5-DmGfrlA or V5-DmGfrlAb (4th and 5th lanes). Equal loading of RET was verified by probing the blot with RET antibody (B, lower panel). Molecular weight markers are shown on the left side of the panels. (PDF) [file pone.0051997.s002.pdf]

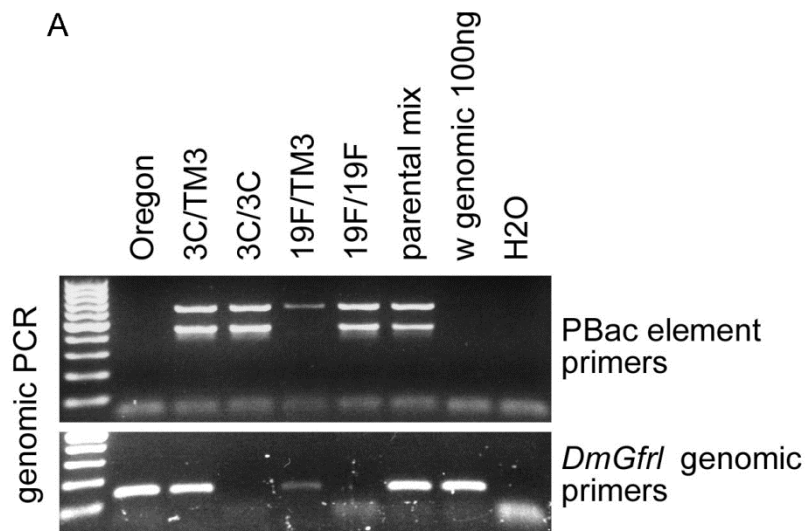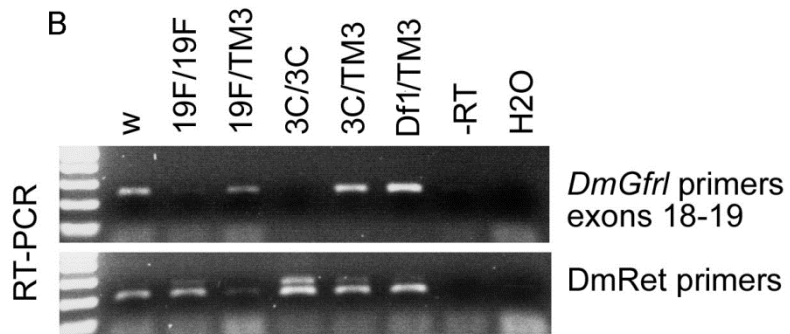

Supplement: Figure S3 — Genotyping and verification of loss of DmGfrl expression in FLP deletion flies. (A) Genomic PCR with primers specific for the PBac elements used in the deletion strategy verifies the presence of remnants of both PBac elements on the same chromosome, indicating correct excision (A, upper panel). Genomic DNA from homozygous and heterozygous adults from two independent deletion lines (3C and 19F) was isolated and subjected to PCR analysis. Oregon and w- were used as negative controls (1st and 7th lanes) and a mixture of genomic DNA from the parental PBac lines as a positive control (6th lane). PCR with DmGfrl genomic primers located within the deletion (A, lower panel) verifies loss of the genomic region in homozygous flies (3rd and 5th lanes). (B) RT-PCR analysis verifies loss of DmGfrl mRNA expression, but not DmRet expression, in flies homozygous for the deletion alleles (2nd and 4th lanes). Expression is detected w- flies (1st lane) and in flies heterozygous for the deletion alleles (3rd and 5th lanes) or for a genomic deficiency (6th lane). (PDF) [file pone.0051997.s003.pdf]

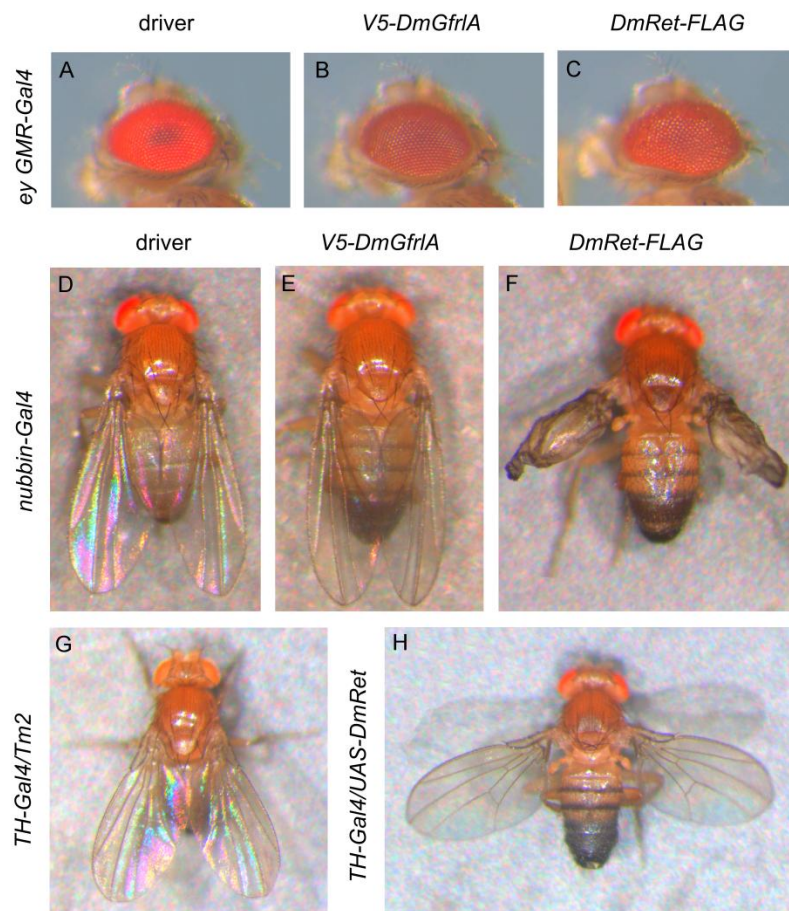

Supplement: Figure S4 — Phenotypes caused by in vivo DmGfrl and DmRet misexpression. (A–H) Newly eclosed adult flies (non-balanced and balanced, when applicable) from the crosses were counted and inspected. The V5-DmGfrl transgene did not cause gross developmetal lethality or apparent adult phenotype when driven with tubulin, da, elav (data not shown), Ey GMR (B) or nubbin (E) drivers. DmRet-FLAG driven with tubulin driver caused embryonic lethality (data not shown), but, surprisingly, no gross lethality with da, elav (data not shown) or TH (H). Driving DmRet expression with the eye-specific Ey GMR (C), wing-specific Nub (F) and tyrosine hydroxylase expressing cell-specific TH (H) drivers caused rough eye, malformed wing and perpendicular wing position, respectively. Co-expression of both receptors in the eye (Ey GMR-Gal4) did not modify the DmRet-induced rough eye phenotype (data not shown). Furthermore, co-expression of both receptors in the nervous system (elav-Gal4) did not cause any obvious synthetic phenotypes (data not shown). (PDF) [file pone.0051997.s004.pdf]

Effect of *DmRet* deficiency on *DmGfrl* mutant male fertility

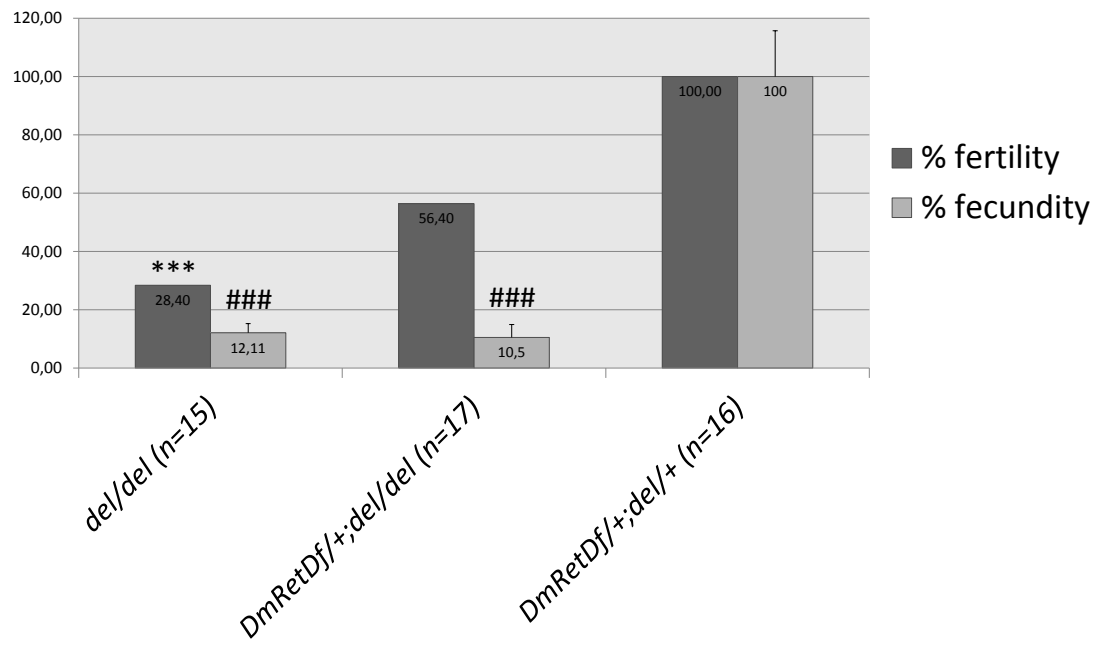

Supplement: Figure S5 — Effect of DmRet deficiency on DmGfrl mutant male fertility. The effect on male fertility of DmRet genomic deficiency (DmRetDf) in combination with the DmGfrl mutant allele (del) was quantified as described in Materials and Methods. The fertility and fecundity of double heterozygous (DmRetDf/+;del/+) control males was set as 100%. The del/del genotype showed statistically highly significantly reduced fertility as compared to the control genotype (non-parametric ANOVA, Kruskall-Wallis with Dunn’s posthoc test, p = 0.0008). The fecundity of both the del/del and the DmRetDf/+;del/del genotypes was statistically highly significantly reduced as compared to the control genotype (one-way ANOVA with Bonferroni’s posthoc test, p<0.0001). In contrast, there was no statistically significant difference in either fertility or fecundity between the del/del and DmRetDf/+;del/del genotypes, which indicates that DmRet heterozygosity did not affect the fertility of DmGfrl mutant males. Error bars represent standard error of mean (SEM). (PDF) [file pone.0051997.s005.pdf]
