# Supplementary material for: Characterization of Drosophila GDNF Receptor-Like and Evidence for Its Evolutionarily Conserved Interaction with Neural Cell Adhesion Molecule (NCAM)/FasII
Source: PLoS One. 2012 Dec 20;7(12):e51997. doi: 10.1371/journal.pone.0051997 (PMC3527400; doi:10.1371/journal.pone.0051997)
Supplement: Table S1 — Genomic locations and features of DmGfrl exons on 3R:16310273-16204718. (PDF) [file pone.0051997.s006.pdf]

Table S1. Genomic locations and features of *DmGfrl* exons on 3R:16310273-16204718.

exon	present in trascripts	coding (yes/no)	note	encodes (parts of)	upper coordinate	lower coordinate	5' end sequence	3' end sequence
1	B, Bb, D	no			16310273	16309697	GTTACAGCAACCAAAACGCG	ATCGCCACCGCTAGCCATGT
2	B, Bb, D	no			16307352	16306415	GTGAACTGTGAGGCAAACAA	ATGTCGCTGATTAATTCCAA
3	B, Bb, D	yes	Kozak/ATG	signal sequence	16290839	16290727	ATTTCAGCTGAACAGTAGAC	ATCGGAATTTTTTATTTAGGT
4	A, Ab	no			16275412	16275166	AACAGTTTTCGACTGAGACTC	AAGCTGTAAATCATAAAGTG
5	A, Ab	yes	Kozak/ATG	signal sequence	16263856	16263401	CAAGCGAAAGTATTGCAGTT	TGGCCATCGGACGATCGGGT
6	A, Ab, B, Bb, D	yes	stop for transcript D after skipping of 3' splice site	domain 0	16236927	16236804	GGTTCCACCATTAAGGGCGT	CGTCTGCGGACCCATTCCAG
7	A, Ab, B, Bb, D	yes		domain 0	16229125	16228946	CATTTGCAGTGTCCTGCAGC	CGGATTTGTGCTGAAGAAAG
8	A, Ab, B, Bb, D	yes		domain 1	16228884	16228734	AGCCGAAAAGGATCCGTAC	AATGCAAAATGGAAAACAGG
9	A, Ab, B, Bb, D	yes		domain 1	16227031	16226896	CAGGGACGCCTGCCACGACT	CACAATCCTTGCGTGGGTAAG
10	C	no			16225502	16225080	GGAGGACGAGGCCAATGAG	GGGTCCCGCACCCACGATTCC
11	C	yes	Kozak/ATG?		16224349	16224246	CACTGGCACCAAAATGCACA	CTCCATGGACGAACGAGGTG
12	Ab, Bb	yes		N-glyc site	16219722	16219387	GTAAGAATATTGGGCATGGA	GCCGGTGTTTACTACCAAAGT
13	A, Ab, B, Bb, C	yes			16216535	16216480	GTGGTCAGGATGTCGAAGTT	ATAATCAAGCAACACTTTCAG
14	A, Ab, B, Bb, C	yes		domain 2	16216155	16215968	CAGAGTACCTGTCACACGGC	ACATCGCCTTTTGTCTATGCAA
15	A, Ab, B, Bb, C	yes		domain 2/3, heparin binding site	16215878	16215684	AAAAACAAGCAGCCAGCAG	AAGGAGGACCGCGAGTGCAG
16	A, Ab, B, Bb, C	yes		domain 3	16213349	16213141	CAGATTAAAGTTGGAGTATT	TGTGGATGAACCCCTGTGTGG
17	A, Ab, B, Bb, C	yes		O-glyc sites	16213005	16212780	GTTGAGGCTCAAAAGGACTT	TCTTCAAGGACGTAATAACGG
18	A, Ab, B, Bb, C	yes		O-glyc sites	16211701	16211373	GCAAAAGAGAAATCGGAGC	ACAACAACACAGCCGCCAAG
19	A, Ab, B, Bb, C	yes			16209590	16209526	GAAACTGCGTTGTCCAGCGA	GGGAAGGCAGCAGCAAGCGA
20	A, Ab, B, Bb, C	yes			16209055	16208830	ATCTACTCCTTGGACGACGT	GGGAGTACATTCTGCCAGGTG
21	A, Ab, B, Bb, C	yes		N-glyc site	16208014	16207890	GGATATTCCTGCTGCTCGGA	ACCACATACATCGATAATCAG
22	A, Ab, B, Bb, C	yes		N-glyc site	16207794	16207686	ACGCAGTGCACCCTCACGCT	TTGAGCTGCTGCGTAAGGAGA
23	A, Ab, B, Bb, C	yes	includes stop codon and 3'UTR	GPI anchoring site	16207298	16204718	AGGACGAGTGCACCGCGAT	CGTTAAAGTAAACTGATAAAT
